# PERK‐Mediated Cholesterol Excretion from IDH Mutant Glioma Determines Anti‐Tumoral Polarization of Microglia

**DOI:** 10.1002/advs.202205949

**Published:** 2023-05-11

**Authors:** Tao Wang, Yunxia Zhou, Yunping Fan, Hao Duan, Xiaoyu Guo, Jinlong Chang, Youheng Jiang, Changxue Li, Zhang Fu, Yunfei Gao, Xiaoran Guo, Kastytis Sidlauskas, Zhenqiang He, Clive Da Costa, Xia Sheng, Dinglan Wu, Jinqiu Yuan, Huiliang Li, Yulong He, Yonggao Mou, Ningning Li

**Affiliations:** ^1^ Tomas Lindahl Nobel Laureate Laboratory The Seventh Affiliated Hospital of Sun Yat‐sen University Shenzhen Guangdong 518107 China; ^2^ Department of Otolaryngology The Seventh Affiliated Hospital of Sun Yat‐sen University Shenzhen Guangdong 518107 China; ^3^ Department of Neurosurgery State Key Laboratory of Oncology in South China & Collaborative Innovation Center for Cancer Medicine Sun Yat‐sen University Cancer Centre Guangzhou Guangdong 510060 China; ^4^ Barts Cancer Institute Queen Mary University of London London London EC1M 6BQ United Kingdom; ^5^ Biological Research Facility The Francis Crick Institute London London NW1 1AT United Kingdom; ^6^ Ministry of Education Key Lab of Environment and Health School of Public Health Tongji Medical College Huazhong University of Science and Technology Wuhan Hubei 430030 China; ^7^ Shenzhen Key Laboratory of Viral Oncology The Clinical Innovation & Research Centre (CIRC) Shenzhen Hospital Southern Medical University Shenzhen Guangdong 510086 China; ^8^ Clinical Research Center Big Data Center The Seventh Affiliated Hospital of Sun Yat‐sen University Shenzhen Guangdong 518107 China; ^9^ Wolfson Institute for Biomedical Research Division of Medicine Faculty of Medical Sciences University College London London London WC1E United Kingdom; ^10^ China‐UK Institute for Frontier Science Shenzhen Guangdong 518000 China; ^11^ Guangdong Provincial Key Laboratory of Digestive Cancer Research The Seventh Affiliated Hospital of Sun Yat‐sen University Shenzhen Guangdong 518107 China

**Keywords:** cholesterol, glioma, glioma‐associated microglia/macrophages, tumor microenvironment

## Abstract

Isocitrate dehydrogenase (IDH) mutation, a known pathologic classifier, initiates metabolic reprogramming in glioma cells and has been linked to the reaction status of glioma‐associated microglia/macrophages (GAMs). However, it remains unclear how IDH genotypes contribute to GAM phenotypes. Here, it is demonstrated that gliomas expressing mutant IDH determine M1‐like polarization of GAMs, while archetypal IDH induces M2‐like polarization. Intriguingly, IDH‐mutant gliomas secrete excess cholesterol, resulting in cholesterol‐rich, pro‐inflammatory GAMs without altering their cholesterol biosynthesis, and simultaneously exhibiting low levels of tumoral cholesterol due to expression remodeling of cholesterol transport molecules, particularly upregulation of ABCA1 and downregulation of LDLR. Mechanistically, a miR‐19a/LDLR axis‐mediated novel post‐transcriptional regulation of cholesterol uptake is identified, modulated by IDH mutation, and influencing tumor cell proliferation and invasion. IDH mutation‐induced PERK activation enhances cholesterol export from glioma cells via the miR‐19a/LDLR axis and ABCA1/APOE upregulation. Further, a synthetic PERK activator, CCT020312 is introduced, which markedly stimulates cholesterol efflux from IDH wild‐type glioma cells, induces M1‐like polarization of GAMs, and consequently suppresses glioma cell invasion. The findings reveal an essential role of the PERK/miR‐19a/LDLR signaling pathway in orchestrating gliomal cholesterol transport and the subsequent phenotypes of GAMs, thereby highlighting a novel potential target pathway for glioma therapy.

## Graphical abstract

The authors demonstrate that excessive cholesterol excreted from Isocitrate dehydrogenase (IDH) ‐mutant glioma cells, as a metabolic messenger, determines M1‐like polarization of glioma‐associated microglia/macrophage (GAMs). This study reveals that the remodeling of GAM phenotypes results from the alteration of tumoral cholesterol homeostasis via IDH mutation‐induced PERK activation, highlighting PERK as a promising therapeutic target for the treatment of glioma.

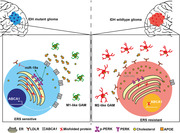

## 1 Introduction

Glioma is the most common type of intracranial tumor, which carries a poor prognosis and leads to devastating clinical outcomes. Currently, there is a lack of available and effective targeted therapies for glioma. Recent research suggests that the rapid proliferation of glioma cells relies heavily on exogenous cholesterol supply instead of de novo cholesterol biosynthesis.^[^ ^1^ ^]^ Studies have shown that inhibiting cholesterol uptake by blocking the low‐density lipoprotein receptor (LDLR) or increasing cholesterol export by upregulating ATP binding cassette subfamily A member 1 (ABCA1) can significantly attenuate cell proliferation and induce cell death in glioma models in vitro and in vivo.^[^ ^1^, ^2^ ^]^ Therefore, targeting the conserved vulnerability of cholesterol metabolism in glioma presents a promising therapeutic avenue.

Isocitrate dehydrogenase (IDH) mutation is a well‐known favorable prognostic biomarker and thereby a molecular classifier, frequently observed in low‐grade glioma and secondary glioblastoma (GBM).^[^ ^3^, ^4^ ^]^ IDH is a rate‐limiting enzyme in the Krebs cycle, and its mutations lead to an ensemble of cellular reprogramming events, affecting glucose, lipid, and amino acid metabolism in several cancer types.^[^ ^5^, ^6^, ^7^ ^]^ A recent study reported that IDH mutation alters cholesterol uptake and secretion in glioma cells, resulting in reduced intracellular cholesterol compared to IDH wild‐type cells,^[^ ^8^ ^]^ suggesting distinct traits of cholesterol metabolism between IDH genotypes. Emerging evidence suggests that in addition to fueling cancer cell growth, cholesterol metabolism plays a crucial role in the remodeling of tumor‐associated macrophages (TAMs).^[^ ^9^, ^10^ ^]^ TAMs account for 30–50% of the total cell population in the tumor microenvironment (TME).^[^ ^11^, ^12^, ^13^, ^14^ ^]^ Recently, single‐cell RNA sequencing (scRNA‐seq) on clinical glioma samples suggested an inextricable correlation between IDH genotypes and distinct functional phenotypes of glioma‐associated microglia/macrophages (GAMs).^[^ ^13^, ^15^ ^]^ Specifically, anti‐tumoral (M1‐like) status is associated with mutant IDH (IDHmt), whereas pro‐tumoral (M2‐like) status is associated with wild‐type IDH (IDHwt).^[^ ^16^, ^17^ ^]^ However, it remains unclear whether IDH genotypes could definitively determine distinct GAM phenotypes, which may be mediated via cholesterol as a metabolic messenger.

The expression of LDLR is tightly regulated by two classical transcriptional factors responsible for cholesterol homeostasis: sterol regulatory element binding transcription factor 2 (SREBP2) at the transcriptional level^[^ ^18^ ^]^ and nuclear receptor subfamily 1 group H member 3 (NR1H3), also known as Liver X receptor‐*α* (LXR*α*), at the post‐translational level.^[^ ^19^ ^]^ Activating LXR through LXR agonists or cholesterol metabolites accelerates LDLR degradation by ubiquitination.^[^ ^1^, ^2^ ^]^ Moreover, LXR is known to upregulate the transcription of ABCA1, promoting the efflux of intracellular cholesterol.^[^ ^1^, ^2^ ^]^ Suppression of LXR in glioma leads to a decline in ABCA1 expression, although LDLR levels remain high.^[^ ^1^, ^2^, ^8^ ^]^ MicroRNAs (miRNAs), small non‐coding RNAs typically consisting of 21–23 base pairs in length, play a critical role in post‐transcriptional regulation of gene expression.^[^ ^20^ ^]^ Several studies have shown that in glioma, three highly expressed miRNAs (miR‐26,^[^ ^21^ ^]^ miR‐33,^[^ ^22^ ^]^ and miR‐148a^[^ ^23^ ^]^) inhibit ABCA1 expression.^[^ ^24^, ^25^, ^26^ ^]^ However, the mechanism by which LDLR is regulated at the post‐transcriptional level remains largely unknown. Deciphering and decoding such a fundamental mechanism in glioma would be of biological and clinical significance.

The endoplasmic reticulum (ER) is a central location for cholesterol metabolism, with several associated proteins, such as 3‐hydroxy‐3‐methylglutaryl‐CoA reductase (HMGCR), 3‐hydroxy‐3‐methylglutaryl‐CoA synthase 1 (HMGCS1), and sterol‐regulatory element binding proteins (SREBPs), anchored to the ER membrane. Disruption of ER homeostasis can trigger ER stress (ERS), which has a significant impact on cholesterol metabolism.^[^ ^27^, ^28^ ^]^ PERK (eukaryotic translation initiation factor 2 alpha kinase 3) serves as a central sensor of ERS and activates ATF4 (activating transcription factor 4)‐mediated gene expression.^[^ ^29^, ^30^ ^]^ Dadey et al. have demonstrated that activation of the PERK/ATF4 axis leads to decreased proliferation and increased apoptosis of GBM cells in response to stimuli such as irradiation.^[^ ^31^ ^]^ Our previous work, and that of others, has shown that IDH mutation enhances the sensitivity of glioma to ERS, resulting in apoptosis through the activation of the PERK/ATF4 arm.^[^ ^32^, ^33^ ^]^ Furthermore, Lita et al. have reported that IDH mutation induces ER dilation, dysfunction of lipid metabolism, and apoptosis of glioma cells.^[^ ^34^ ^]^ Fusakio et al. have suggested ATF4 is involved in cholesterol homeostasis via promoting ABCA1 transcription in the liver.^[^ ^35^ ^]^ These observations suggest that there might be a regulatory link between IDHmt‐induced PERK/ATF4 activation and cholesterol metabolism in glioma. However, the precise mechanism by which the PERK/ATF4 axis mediates cholesterol homeostasis and orchestrates the phenotypes of GAMs remains poorly understood and requires further investigation.

In this study, we find that IDH mutation in glioma cells induces cholesterol efflux, resulting in M1‐like, anti‐tumoral polarization of GAMs in a PERK‐dependent manner. Specifically, PERK activation remodels cholesterol transport in IDHmt glioma cells by downregulating LDLR and upregulating ABCA1. Furthermore, we show that CCT020312 (CCT), a synthetic PERK inducer, effectively promotes cholesterol export in IDHwt glioma and activates M1‐like polarization of GAMs. Our findings offer new insights into future anti‐glioma clinical strategies by targeting the IDH/PERK/cholesterol axis alone or in combination with immunotherapy.^[^ ^36^ ^]^

## 2 Results

### 2.1 IDH Genotypes of Gliomas Sculpt Distinct Phenotypes of GAMs

Recent studies suggest a noteworthy correlation between IDH genotypes of gliomas and the phenotypic diversity of GAMs in the pertinent TME,^[^ ^15^ ^]^ implying a potential role of IDH mutations in the interplay between gliomas and their associated GAMs. To investigate this relationship further, we performed a comprehensive analysis of published datasets of single‐cell RNA sequencing (scRNA‐seq)^[^ ^13^, ^37^ ^]^ to identify the proportion, polarization, and function of GAMs in the TME based on tumoral IDH classification. Our analysis of immune cell infiltration revealed that GAMs were the predominant cell population in lower‐grade gliomas, despite distinct immune‐layouts of the TME between IDHwt and IDHmt gliomas (Figure S1A, Supporting Information). We also found that IDHmt gliomas had a higher proportion of GAMs with an M1‐like phenotype (IDHmt: IDHwt = 90.62%: 75.86%) (Figure S1B, Supporting Information), as supported by an augmented proinflammatory signaling consisting of GAM‐associated cytokines and receptors, such as TNF, TLR2, and TNFRSF1B (Figure S1C, Supporting Information). Furthermore, we observed that GAMs in IDHmt gliomas exhibited a significant increase in the expression of M1‐associated markers and cytokines, such as CD86 and IL1B, and a significant decrease in M2‐associated genes, such as CD163 and CD206, relative to IDHwt counterparts (Figure S1D, Supporting Information). In keeping with these findings, our TCGA (The Cancer Genome Atlas) bulk RNA‐seq analysis confirmed GAMs as the predominant immune cell type in both subtypes of gliomas (Figure S1E, Supporting Information) and M1‐like polarization as an advantageous phenotype of GAMs in IDHmt glioma (Figure S1F, Supporting Information). In addition, the Kaplan‐Meier analysis showed that IDHmt glioma patients with a higher M1 score had a favorable prognosis (Figure S1G, Supporting Information).

**Figure 1:**
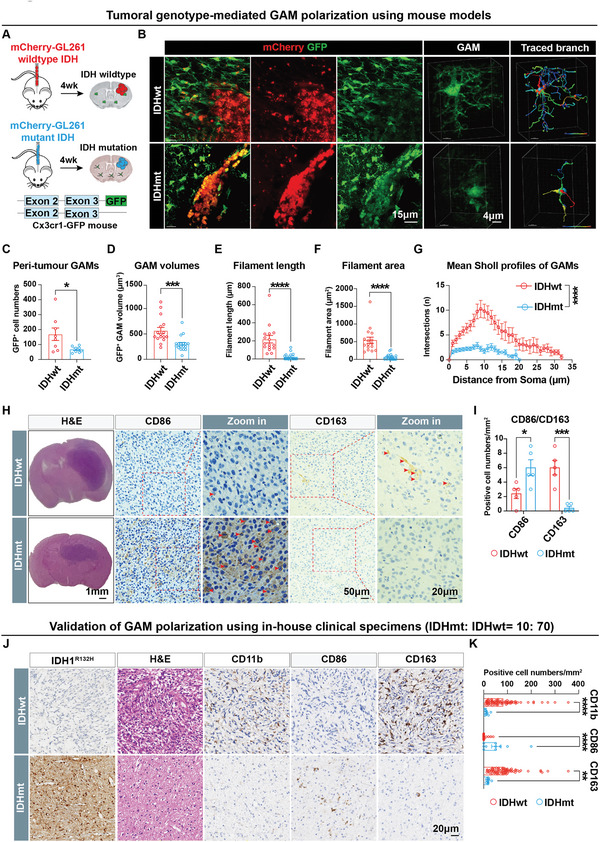
IDH genotypes in gliomas determine the polarization of glioma‐associated microglia/macrophages (GAMs). A) Schematics of the generation of syngeneic glioma mouse models through stereotaxic injection of different IDH genotypes of GL261 cells into Cx3cr1‐GFP mice. B) Representative images of immunofluorescence (IF) staining shows mCherry‐labeled glioma cells and GFP‐labeled microglia in the syngeneic models in the left panels. 3D image reconstructions of GAMs and representative traced branch images of GAMs are shown in the right panels. C) Quantification of numbers of GFP^+^ cells in the murine glioma samples. Tumor sample number: IDHmt: IDHwt = 3: 3, and number of views (20X objective): IDHmt: IDHwt = 8: 8. D–G) Quantification of volumes (D), filament length (E) and area (F), and mean Sholl profiles (G) of individual GFP^+^ cells in the murine glioma samples via 3D surface analysis. Tumor sample number: IDHmt: IDHwt = 3: 3, and GFP^+^ cell number (63X objective): IDHmt: IDHwt = 18: 16. H,I) Representative images of hematoxylin and eosin (H&E) and immunohistochemistry (IHC) staining show expression of CD86 and CD163 in GL261‐induced syngeneic glioma mouse models (H) and associated quantification of marker expression (I) (IDHmt: IDHwt = 5: 5). J,K) Representative images of IHC staining show expression of IDH1^R132H^, CD11b, CD86, and CD163 in serial sections of human glioma samples (J) and associated quantification of marker expression (K) (IDHmt: IDHwt = 10: 70). Data are shown as means ± SEM. Statistical significance is determined using the two‐tailed Student's t‐test, *p< 0.05; **p< 0.01; ***p< 0.001; ****p< 0.0001.

To investigate the causal relationship between IDH genotypes and GAM phenotypes, we designed rigorous in vivo experiments to determine whether IDH1^R132H^ mutation and archetypal IDH could induce opposite GAM phenotypes. We established a syngeneic glioma model by stereotaxically implanting mCherry‐labeled GL261 murine glioma cells into a mouse with a Cx3cr1‐GFP allele that labels microglial cells with high fidelity (**Figure** **1**A). Morphological differences in GAMs were observed between IDHwt and IDHmt gliomas. To better visualize complex structures of GAMs and to quantify potential anomalies, we constructed three‐dimensional (3D) surface renders of individual GFP^+^ cells using Imaris (Figure 1B). First, we counted the number of individual GFP^+^ renders adjacent to glioma lesions and found that IDHwt glioma recruited a relatively higher number of peri‐tumoral GAMs than IDHmt gliomas (Figure 1C). Then, we isolated individual GFP^+^ surface renders and observed that the volumes of GAMs in IDHmt gliomas were significantly smaller than their IDHwt counterparts (Figure 1D). As activated microglia tend to reduce the complexity of the foot process, we assessed the length and surface area of the foot process of GFP^+^ GAMs, which were significantly decreased in IDHmt gliomas (Figure 1E,F). Furthermore, the Sholl profiling analysis, which calculates the number of foot processes at each ring from the cell soma, revealed that the complexity of GAMs in IDHmt gliomas was also profoundly reduced (Figure 1G). Overall, our results suggest that GAMs in IDHmt gliomas exhibit significant morphological changes, including smaller soma and less complexity, compared to IDHwt counterparts.

Further, we sought to investigate whether the phenotypic differences in GAMs elicited by IDH mutation were associated with their polarization status. To this end, we conducted immunohistochemistry (IHC) staining on the GL261 syngeneic gliomas samples and observed that GAMs in IDHmt gliomas exhibited high expression levels of CD86, a marker of M1‐like polarization, but extremely low levels of CD163, a marker of M2‐like polarization, relative to GAMs in IDHwt gliomas (Figure 1H,I). To validate our findings, we used an intrinsic glioma mouse model by stereotaxic injection of lentivirus carrying pTomo‐Ras‐sip53^[^ ^38^, ^39^ ^]^ (Figure S2A, Supporting Information). Immunofluorescence staining revealed a significant increase in CD163^+^ microglia in the archetypal IDH glioma. Strikingly, an additional IDH^R132H^ mutation reversed the M2‐like phenotype to M1, resulting in a significant enhancement in CD86^+^ microglia (Figure S2B,C, Supporting Information).

Finally, to confirm the link between IDH genotypes and GAM phenotypes, we evaluated M1/M2 associated markers in serial sections of clinical specimens (IDHmt: IDHwt = 10: 70) obtained from our institutions. Consistent with findings in the syngeneic glioma mouse model, our IHC staining showed that IDHwt gliomas recruited significantly more CD11b^+^ GAMs than IDHmt counterparts. Notably, IDHmt specimens exhibited significantly enhanced CD86^+^ GAMs, while IDHwt samples showed markedly increased CD163^+^ GAMs, indicative of M1‐ and M2‐like phenotypes, respectively, in these two distinct subtypes of tumor entities (Figure 1J,K).

Collectively, our results suggest that IDH mutation is a crucial driver for microglial M1‐like polarization, distinguishing it as a separate entity from IDHwt glioma that predominantly maintains M2‐like GAMs. These in vivo observations are in line with our bioinformatics analysis.

### 2.2 Excess Extracellular Cholesterol Released by Mutant IDH Glioma Cells Determines Microglial M1‐like Polarization

**Figure 2:**
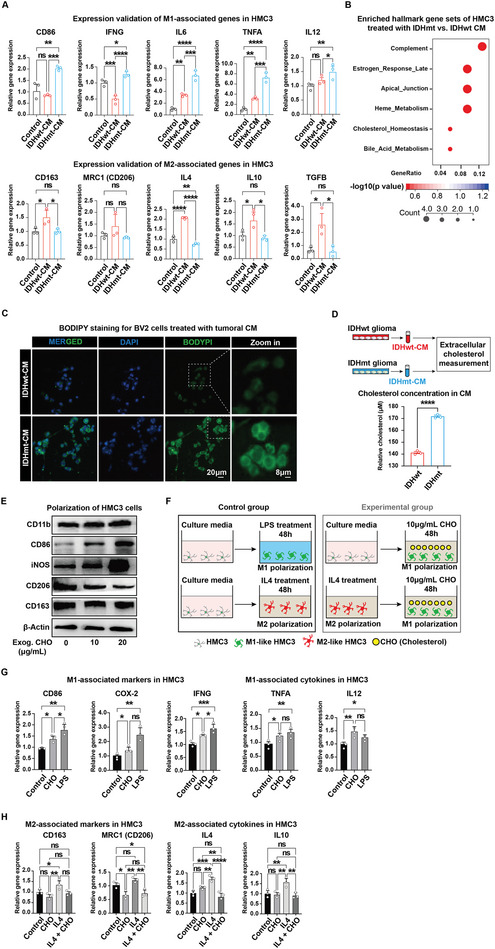
Cholesterol released by glioma cells induces microglial M1‐like polarization. A) Quantitative reverse transcription PCR (RT‐qPCR) analysis of polarization‐associated gene expression levels in HMC3 cells treated with normal growth medium or conditioned medium (CM) collected from IDHwt or IDHmt glioma cells (i.e., IDHwt‐CM or IDHmt‐CM), respectively. ACTB is used as an internal control. Data are presented as means ± SEM (*n* = 3). Statistical significance is determined by the one‐way ANOVA, ns, *p*> 0.05; *p< 0.05; **p< 0.01; ***p< 0.001; ****p< 0.0001. B) Hallmark enrichment analysis of differentially expressed genes with adjusted *p*‐values using RNA‐seq data from HMC3 cells treated with IDHwt‐CM versus IDHmt‐CM. C) Representative images of BODIPY staining in BV2 cells treated with IDHwt‐CM or IDHmt‐CM. D) Schematic showing measurement of extracellular cholesterol released from glioma cells with different IDH genotypes. Data are shown as the means ± SEM (IDHmt: IDHwt = 3: 3). Statistical significance is determined by the two‐tailed Student's t‐test, ****p< 0.0001. E) Western blotting analysis of the protein expression levels of GAM‐associated markers, CD11b, CD86, iNOS, CD206, and CD163 in HMC3 cells treated with a cholesterol concentration gradient. *β*‐Actin is used as a loading control. F) Schematic showing HMC3 cells treated with normal growth medium (control), and with or without additional 10 µg mL^−1^ cholesterol solution. HMC3 cells were treated with 100 ng mL^−1^ of LPS as an M1‐like polarization control or 40 ng mL^−1^ of IL4 as an M2‐like polarization control. G,H) RT‐qPCR analysis of expression levels of (G) M1‐ and (H) M2‐associated markers and cytokines in HMC3 cells upon cholesterol challenge. ACTB is used as an internal control. Data are presented as means ± SEM (*n* = 3). Statistical significance is determined by the one‐way ANOVA, ns, *p*> 0.05; *p< 0.05; **p< 0.01; ***p< 0. 001; ****p< 0.0001.

Cytokines and chemokines are well‐established mediators of crosstalk between tumors and TME.^[^ ^40^, ^41^ ^]^ To investigate whether different IDH genotypes modulate GAM polarization through such mechanisms, we tested the functional changes of microglia treated with tumoral conditioned medium (CM) collected from the different genotypes of glioma cell cultures. Initially, we established three glioma cell lines stably expressing the IDH1^R132H^ mutation based on wild‐type A172, SF295, and U87 cells, and confirmed enhanced D‐2‐hydroxyglutarate (D2HG) production in the IDHmt cells (Figure S3A,B, Supporting Information), which reliably recapitulated the biological outputs of clinical IDH mutations, including slower proliferation, migration, invasion, and colony formation (Figure S3C–F, Supporting Information). Subsequently, we cultured HMC3 microglial cells using CM collected from U87 cells with or without IDH1 mutation (Figure S4A, Supporting Information) and then performed RNA sequencing. Uniform manifold approximation and projection (UMAP) analysis identified three distinct populations of microglia treated with standard medium or tumoral CM (IDHwt‐CM versus IDHmt‐CM) (Figure S4B,C, Supporting Information). Visualization and analysis of gene expression data revealed that M2 polarization‐associated gene sets were enriched in IDHwt‐CM‐treated HMC3 cells, while M1 polarization‐associated gene sets were enriched in IDHmt‐CM‐treated cells (Figure S4D,E, Supporting Information). RT‐qPCR analysis confirmed that HMC3 cells treated with IDHmt‐CM highly expressed M1‐associated markers and cytokines, including CD86, IFNG, IL6, TNFA, and IL12 (**Figure** **2**A, upper panel). In contrast, HMC3 cells treated with IDHwt‐CM significantly upregulated M2‐associated markers and cytokines, such as CD163, MRC1 (CD206), IL4, IL10, and TGFB1 (Figure 2A, lower panel). Taken together, these in vitro findings recapitulated the capacity of IDH genotypes to shape the discrete polarization of microglia, in keeping with our in vivo data. Thus, our in vitro culture assay provides a robust means of investigating the underlying mechanism for the GAM phenotype changes in response to messenger molecules.

Notably, our RNA‐seq bubble plot revealed dysregulation of cholesterol homeostasis in HMC3 cells treated with IDHmt‐CM (Figure 2B). Gene set enrichment analysis (GSEA) suggested elevated cholesterol storage resulting from IDHmt‐CM treatment (Figure S4F, Supporting Information). To validate these findings, we utilized boron dipyrromethene (BODIPY) staining, a method that labels cholesterol in its native state,^[^ ^9^ ^]^ and observed a significant accumulation of cholesterol on the plasma membrane of BV2 microglial cells treated with IDHmt‐CM (Figure 2C). Intriguingly, despite the observed increase in intracellular cholesterol levels, the transcriptomic profiling of differentially expressed genes did not show significant changes in genes related to cholesterol synthesis, uptake, esterification, and export (Figure S4G, Supporting Information), indicating that the cholesterol production capacity of microglial cells treated with different tumoral CM was unaltered. Therefore, we sought to determine the potential source of the elevated cholesterol. By assessing cholesterol abundance in the tumoral CM, we found that the cholesterol concentration in the IDHmt‐CM was significantly higher than that in the IDHwt‐CM (Figure 2D), implying that the observed intracellular cholesterol accumulation was likely due to the increased import of cholesterol into microglial cells from the extracellular medium.

To investigate whether cholesterol per se could act as a determinant for the phenotypic reprogramming of microglia, we cultured HMC3 microglial cells in normal growth medium (DMEM with 10% FBS containing approximately 2 µg mL^−1^ cholesterol) supplemented with or without exogenous cholesterol. Immunoblotting analysis showed that increasing concentrations of exogenous cholesterol (0, 10, and 20 µg mL^−1^) resulted in a significant upregulation of CD86 and iNOS expression but a profound diminution of CD206, although CD163 expression remained unaltered (Figure 2E). These results suggest that cholesterol may play an important role in remodeling microglial polarization by promoting M1‐like but suppressing M2‐like polarization.

To compare the efficiency of cholesterol in inducing microglial polarization with that of lipopolysaccharide (LPS) or IL4 (interleukin 4), known as standards for M1‐ or M2‐like polarization induction, we performed RT‐qPCR analysis on HMC3 and BV2 cell culture in the presence of 10 µg mL^−1^ exogenous cholesterol (Figure 2F). As expected, we found that this exogenous cholesterol concentration significantly upregulated the expression of M1‐associated markers and cytokines, including CD86, COX‐2, IFNG, TNFA, and IL12 in HMC3 cells, which exhibited a pro‐inflammatory state comparable to LPS‐induced M1‐like polarization (Figure 2G). Similarly, cholesterol induced M1‐like polarization in BV2 cells (Figure S5A, Supporting Information). Although cholesterol at the same concentration did not profoundly affect the M2‐like polarization of the HMC3 cells, it significantly suppressed the IL4‐induced M2‐like polarization and predisposed these cells to a phenotype similar to that of the cholesterol‐free control group by dramatically reducing M2‐associated genes, including CD163, MRC1 (CD206), IL4, and IL10 (Figure 2H), which was consistent with the findings for BV2 cells (Figure S5A, Supporting Information). Immunofluorescence staining supported that CD86 was remarkably upregulated and CD206 was reduced following the cholesterol challenge, further confirming the role of cholesterol in shaping the microglial M1‐like phenotype (Figure S5B, Supporting Information).

Taken together, as both exogenous cholesterol and excreted cholesterol from the IDHmt‐CM could trigger M1‐like microglial polarization, we postulate that cholesterol might act as a tumoral metabolic messenger that orchestrates the phenotype of GAMs.

### 2.3 Intracellular Cholesterol in IDHmt Tumoral Parenchyma was Reduced In Vivo and In Vitro

**Figure 3:**
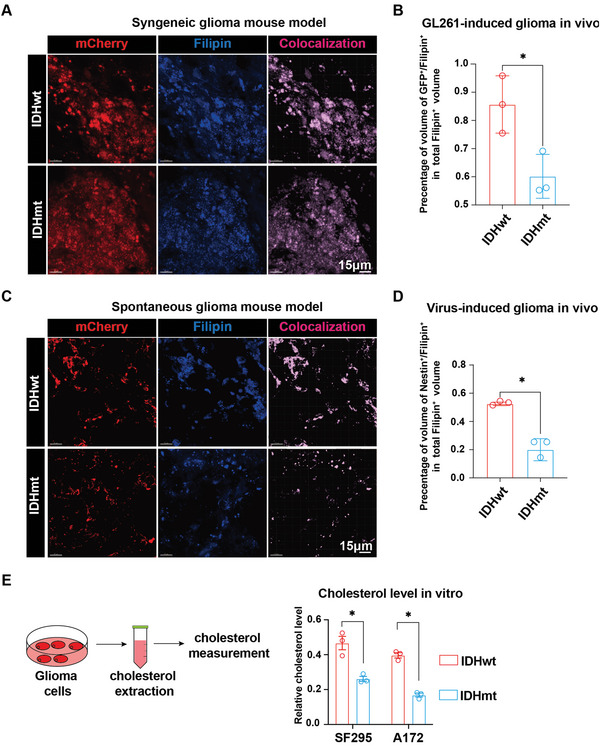
IDH genotypes orchestrate distinct cholesterol abundance in gliomas. A) Representative images of immunofluorescence staining showing mCherry^+^ glioma cells (red), and Filipin staining (blue) in a GL261‐induced syngeneic glioma mouse model. B) Quantification of the percentage of Filipin^+^/mCherry^+^ co‐localized volume in total Filipin^+^ volume in murine glioma samples stratified by different IDH genotypes. C) Representative images of immunofluorescence staining showing Nestin^+^ glioma cells (red) and Filipin staining (blue) in a virus‐induced spontaneous glioma mouse model. D) Quantification of the percentage of Filipin^+^/Nestin^+^ co‐localized volume in total Filipin^+^ volume in murine glioma samples stratified by IDH mutation. E) Schematics of the intracellular cholesterol measurement of glioma cells (left panel) and comparison of intracellular cholesterol concentrations in A172 and SF295 cells with or without an IDH^R132H^ mutation (right panel). Data are shown as means ± SEM (IDHwt: IDHmt = 3: 3). Statistical significance is determined by the two‐tailed Student's t‐test, *p< 0.05.

Seeing as extracellular cholesterol‐mediated GAM polarization may result from tumoral cholesterol origin, we sought to investigate whether glioma cells with different IDH genotypes preserve cholesterol differentially. Filipin, a mixture of antibiotics obtained from *Streptomyces filipinensis*, binds specifically to cholesterol, forming complexes without affecting other molecules and compounds. Therefore, we used Filipin and immunofluorescence staining to visualize and evaluate the relative abundance of cholesterol in the IDHmt versus IDHwt glioma parenchyma of the GL261 syngeneic model. First, we constructed 3D images from the tumor section to identify the co‐localized channel for Filipin^+^ (cholesterol) and mCherry^+^ (glioma cells). Then, by comparing the ratio of Filipin^+^/mCherry^+^ volume to the total Filipin^+^ volume, we quantified the relative abundance of cholesterol in glioma cells. We found that IDHmt glioma cells had a significantly lower cholesterol occupancy than their IDHwt counterparts (**Figure** **3**A,B, IDHwt: IDHmt = 0.8566: 0.6071, *p* = 0.0262). To confirm this observation, we employed a lentivirus‐induced intrinsic glioma mouse model and labeled cholesterol and glioma cells using Filipin and Nestin, respectively. Consistently, we found that IDHmt glioma cells contained significantly less Filipin^+^/Nestin^+^ volumes than IDHwt counterparts, indicating a reduced intracellular cholesterol level in IDHmt tumor parenchyma (Figure 3C,D, IDHwt: IDHmt = 0.52: 0.19, *p* = 0.0012). Furthermore, a colorimetric assay for cholesterol concentration showed that IDHmt SF295 and A172 glioma cells also contained significantly lower intracellular cholesterol than IDHwt cells (Figure 3E). These in vivo and in vitro data led us to focus on cholesterol regulation in glioma cells.

### 2.4 IDH Mutation Results in a Low Abundance of Intracellular Cholesterol in Glioma Cells by Downregulating LDLR and Upregulating ABCA1

To investigate how glioma cells with different IDH genotypes explicitly control their intracellular cholesterol levels, we accessed the CGGA (Chinese Glioma Genome Atlas) RNA‐seq data and conducted GSEA, which suggested enrichment of cholesterol biosynthesis‐ and export‐associated gene sets in IDHmt gliomas compared to IDHwt counterparts (Figure S6A, Supporting Information). To reduce the impact of bulk RNA‐seq on the output of specific cell types, we utilized two published scRNA‐seq datasets (IDHmt: Venteicher et al. 2017;^[^ ^13^ ^]^ IDHwt: Yu et al. 2020^[^ ^37^ ^]^) to identify the significantly enriched hallmark gene sets specifically in glioma cells with different IDH genotypes. A bubble plot revealed that cholesterol homeostasis was notably enriched in IDHwt glioma cells (Figure S6B, Supporting Information). We then compared the transcriptional expression of the genes involved in cholesterol homeostasis, synthesis, and transport. We found that IDHmt cells exhibited downregulation of three key cholesterol synthesis enzymes (HMGCR, HMGC1, and DHCR24), concomitant with augmented expression of cholesterol efflux regulator ABCA1 and reduced expression of the cholesterol uptake gene, LDLR, compared to IDHwt glioma cells (Figure S6C, Supporting Information).

**Figure 4:**
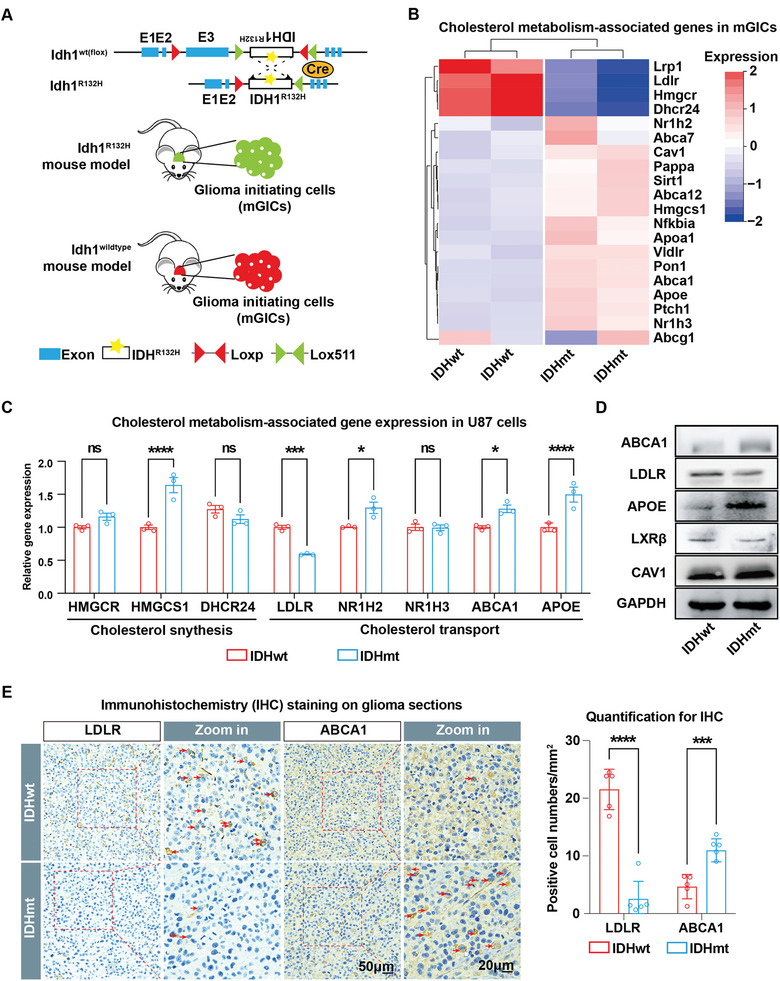
IDH mutation remodels cholesterol transport through increased ABCA1 and declined LDLR. A) Schematics of the generation of genetic mouse models for gliomas with different Idh1 genotypes and the concomitant isolation of mouse glioma initiating cells (mGICs) reported previously.^[^ ^32^ ^]^ B) Heatmap of differentially expressed transcripts from RNA‐seq analysis of mGICs carrying different IDH genotypes. Gene expression data are mean z‐transformed for display and colored red for high expression and blue for low expression. C) RT‐qPCR analysis of expression levels of cholesterol metabolism‐associated genes in IDHwt versus IDHmt U87 cells (IDHmt: IDHwt = 3: 3). GAPDH is used as an internal control. D) Immunoblotting analysis of expression levels of cholesterol transport‐associated regulators in U87 cells infected with Lenti‐control or Lenti‐IDH1^R132H^. GAPDH is used as a loading control. E) Representative images (left panel) and quantification (right panel; IDHmt: IDHwt = 5: 5) of IHC staining showing LDLR and ABCA1 protein levels in gliomas from the genetic mouse models. Red arrowheads denote ABCA1^+^ or LDLR^+^ cells (left panel). Data are shown as means ± SEM. Statistical significance is determined by the two‐tailed Student's t‐test (right panel), ns, *p*> 0.05; *p< 0.05; ***p< 0. 001; ****p< 0.0001.

To illustrate the core impact of IDH mutation on tumoral cholesterol metabolism and to set it apart from various confounding genetic vulnerabilities in clinical samples, we compared our previous mouse models of intrinsic gliomas initiated by the inactivation of tumor suppressor genes Pten and Tp53 in forebrain progenitors, with or without expression of an additional human IDH1^R132H^ mutation.^[^ ^32^ ^]^ The IDH mutation is the sole identifier of the resulting murine glioma‐initiating cells (mGICs), which were isolated from the mouse brain parenchyma (**Figure** **4**A). RNA‐seq analysis of mGICs showed a significant deficit of cholesterol synthesis (Hmgcr and Dhcr24) and uptake (Lrp1 and Ldlr), but a profound increase in cholesterol efflux (Nr1h2, Nr1h3, Abca1, Abca7, Apoa1, Apoe, and Cav1) in IDHmt mGICs relative to the IDHwt control (Figure 4B). Further, using RT‐qPCR, we confirmed significantly downregulated LDLR and upregulated NR1H2, ABCA1, ABCG1, and APOE in human IDHmt U87 glioma cells compared to the IDHwt control (Figure 4C). Consistent with these findings, immunoblotting analysis showed that the exogenous IDH1^R132H^ mutation reduced LDLR expression and enhanced the expression of cholesterol export regulators, ABCA1, CAV1, and APOE, while LXR*β* (NR1H2) remained unchanged (Figure 4D). IHC staining on clinical specimens confirmed a significant reduction in LDLR and a substantial increase in ABCA1 in IDHmt versus IDHwt cohorts (IDHmt: IDHwt = 5: 5) (Figure 4E). Altogether, our results suggest IDH mutation disrupts tumoral cholesterol metabolism, mainly characterized by enhanced efflux through upregulated ABCA1 and reduced influx through downregulated LDLR.

### 2.5 miR‐19a is Significantly Upregulated in IDHmt Glioma and Targets LDLR mRNA for Degradation

**Figure 5:**
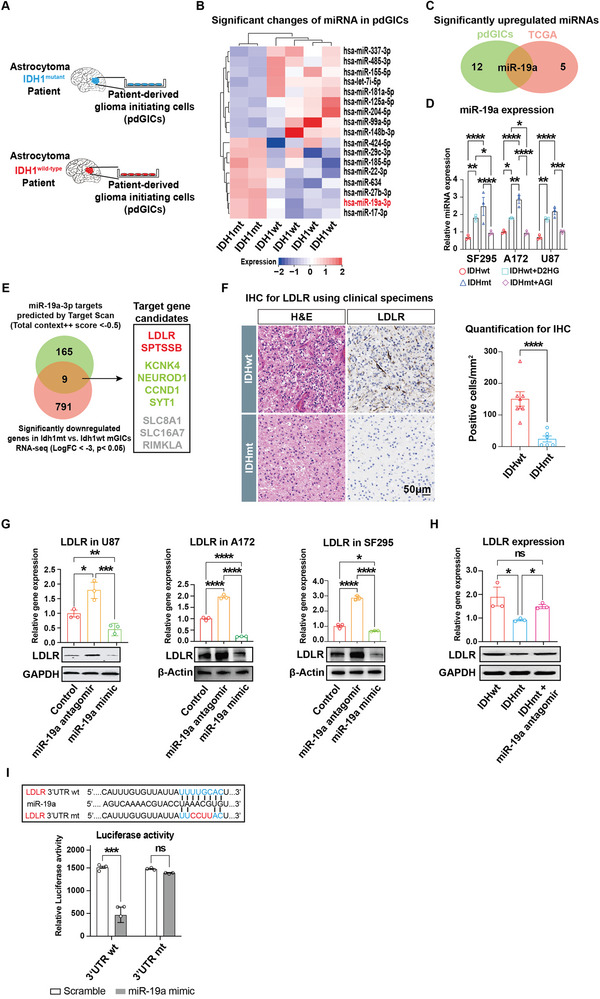
Identification of miR‐19a/LDLR axis in IDH mutant glioma cells. A) Schematic representation of isolation of patient‐derived glioma‐initiating cells (pdGICs). B) Heatmap of significantly differentially expressed microRNAs (miRNAs) between IDHmt and IDHwt pdGICs (IDHmt: IDHwt = 2: 4). Gene expression values are z‐transformed and colored red for high expression and blue for low expression, as indicated in the scale bar. C) Venn diagram showing miR‐19a as the only overlapped miRNA candidate among the significantly upregulated miRNAs in IDHmt pdGICs (in‐house) and gliomas (TCGA) relative to IDHwt counterparts. D) RT‐qPCR analysis of miR‐19a expression levels in three glioma cell lines, SF295, A172, and U87, in the presence and absence of IDH mutation, and with and without additional treatment of 5 µm D2HG in IDHwt cells or 50 nm AG1‐5198 in IDHmt cells for 24 h. RNU6B is used as an internal control. Data are shown as means ± SEM (*n* = 3). Statistical significance is determined by the one‐way ANOVA, *p < 0 .05; **p < 0 .01; ***p< 0. 001; ****p< 0.0001. E) Venn diagram displaying nine overlapped target molecules of miR‐19a using TargetScan prediction and mGICs RNA‐seq results (left panel). Genes correlated with poor prognosis are colored red, genes correlated with favorable survival are green, and genes not correlated with prognosis are grey (right panel). F) Representative images of H&E and IHC staining for LDLR (left panel) and the subsequent quantification of LDLR^+^ cells (right panel) in human glioma specimens. Data are means ± SEM (IDHmt: IDHwt = 6: 8). Statistical significance is determined by a two‐tailed Student's t‐test, ****p< 0.0001. G) RT‐qPCR and immunoblotting analysis of LDLR expression levels in U87, A172, and SF295 glioma cells transfected with control vector, miR‐19a antagomirs, and mimics. Data are mean ± SEM (*n* =  3). Statistical significance is determined by the one‐way ANOVA, ns, *p*> 0 .05; *p< 0 .05; **p< 0 .01; ***p< 0. 001; ****p< 0.0001. GAPDH or *β*‐Actin is used as an internal or loading control. H) RT‐qPCR and immunoblotting analysis of LDLR expression levels in A172 glioma cells carrying an IDH1^R132H^ mutation per se or with exogenous miR‐19a antagomirs relative to archetypal IDH. Data are presented as mean ± SEM (*n* =  3). Statistical significance is determined by the one‐way ANOVA, ns, *p*> 0.05; *p< 0.05. GAPDH is used as an internal or loading control. I) Alignment of miR‐19a binding sequence to the 3′UTR of LDLR. A mutation in the 3′UTR of LDLR is generated in the site complementary to the seed region of miR‐19a. The interaction of miR‐19a with the 3′UTR of LDLR was assessed by measuring the relative luciferase activity of A172 glioma cells transfected with miR‐19a mimics or control. Data are shown as means ± SEM (*n* = 3). Statistical significance is determined by the two‐tailed Student's t‐test, ns, *p*> 0.05; ***p< 0.001.

MiRNAs have been implicated in the precise regulation of cholesterol homeostasis and transport.^[^ ^42^ ^]^ However, the post‐transcriptional profile of LDLR in glioma has not been elucidated yet. To better understand the mechanistic link between miRNAs and cholesterol metabolism in IDHmt versus IDHwt gliomas, we first compared miRNA expression profiles of astrocytoma patient‐derived glioma‐initiating cells (pdGICs) with or without IDH mutations (IDHmt: IDHwt = 2: 4) (**Figure** **5**A). We identified 17 significantly differentially expressed miRNAs (DemiRs), of which eight were upregulated in IDHmt pdGICs, while nine were enriched in the IDHwt counterparts (Figure 5B). After matching against DemiRs in TCGA, miR‐19a was found to be the only upregulated miRNA in IDHmt gliomas (Figure 5C). To investigate whether miR‐19 is a bona fide target regulated by IDH mutation, we treated the three IDHwt glioma cell lines (SF295, A172, and U87) with 5 µm D2HG (a key metabolic product resulting from an IDH mutation) and found a significant induction of miR‐19a. In contrast, a remarkable reduction of miR‐19a was observed in IDHmt cells upon treatment with AGI‐5198 (an inhibitor of IDH1^R132H^) (Figure 5D). These results suggest a regulatory role of IDH mutation in the expression of miR‐19a as a signature miRNA in glioma cells.

To screen for target molecules of miR‐19a relevant to cholesterol metabolism, we performed a TargetScan analysis to predict the downstream targets of miR‐19a (total context^++^ score< −0.5) and compared the results with the significantly downregulated genes in IDHmt mGICs (LogFC< −3, *p*< 0.05) (Figure 5E). We identified 9 candidate targets (Figure 5E), including LDLR and SPTSSB, which are closely correlated with poor clinical prognosis, and four others that are associated with favorable survival outcomes as per TCGA (Figure S7A, Supporting Information). Further analysis using bulk RNA‐seq from public databases (TCGA and CGGA) and scRNA‐seq from published datasets confirmed that LDLR was significantly downregulated in IDHmt gliomas (Figure S7B,C, Supporting Information). IHC labeling of clinical specimens also demonstrated that the expression of LDLR was significantly decreased in IDHmt gliomas (IDHmt: IDHwt = 6: 8) (Figure 5F), indicating a negative association between IDH mutation and LDLR expression.

Further, using a miR‐19a mimic or antagomir (Figure S7D, Supporting Information), our RT‐qPCR and immunoblotting analysis confirmed miR‐19a regulation of LDLR expression in glioma cells, resulting in a highly significant downregulation or upregulation of LDLR, respectively (Figure 5G). Notably, overexpression of miR‐19a in IDHwt glioma cells reduced LDLR expression to a level comparable to that in IDHmt cells (Figure S7E, Supporting Information). In contrast, the miR‐19a antagomir could reverse the intrinsic low levels of LDLR expression in IDHmt cells (Figure 5H). To further validate the direct interaction between miR‐19a and LDLR, we performed a luciferase reporter assay using the 3’ untranslated region (UTR) of LDLR containing a wild‐type or mutated miR‐19a‐5p‐binding site (Figure 5I, upper panel). Our results demonstrated a significant reduction in luciferase reporter activity upon miR‐19a binding to the wild‐type UTR, but not to the mutant control (Figure 5I, lower panel). Collectively, our findings highlight the miR‐19a/LDLR axis as a post‐transcriptional regulatory mechanism of cholesterol transport in IDHmt glioma. This mechanism may provide a possible explanation for cholesterol‐mediated M1‐like microglial polarization.

### 2.6 Down‐Regulation of LDLR Inhibits Cholesterol Uptake, Proliferation, and Invasion of Glioma Cells

**Figure 6:**
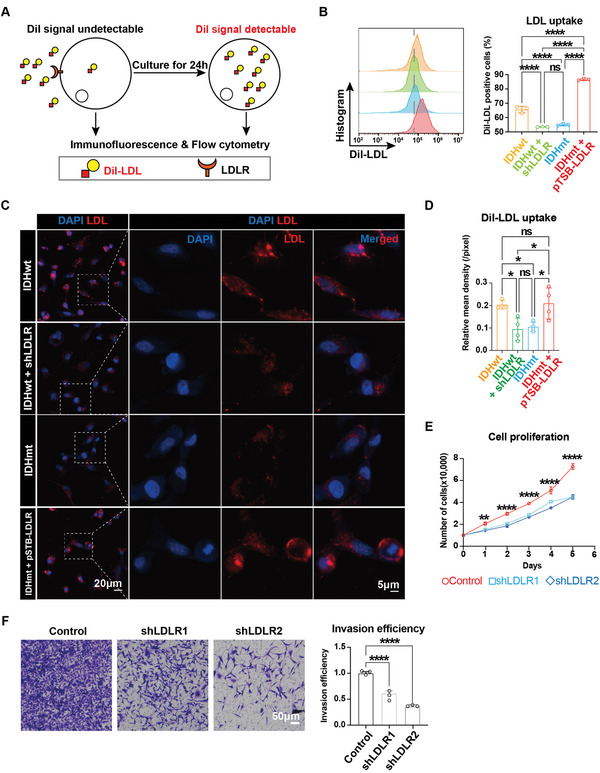
LDLR is essential for cholesterol influx, proliferation, and invasion of glioma cells. A) Schematics of assessment of cholesterol uptake by glioma cells using DiI‐LDL. B) Flow cytometry analysis for DiI^+^ cells indicating the percentage of LDL uptake in U87 cells transfected with control, shLDLR, IDH1^R132H^, or IDH1^R132H^ + pTSB‐LDLR vectors (*n* = 3). C,D) Representative images of immunofluorescence staining (C) of DiI‐LDL uptake in U87 cells transfected with vectors shown in (B), and associated quantification (D) shown as relative mean density (*n* = 4). E) Growth curves for A172 cells infected with control or shLDLR via the CCK‐8 assay (*n*  =  3). F) Representative images (left panel) and analysis (right panel) of the transwell assay for A172 cells infected with control or shLDLR (*n* = 3). Data are shown as means ± SEM. Statistical significance is determined by the one‐way ANOVA, ns, *p*> 0 .05; *p< 0.05; **p< 0 .01; ****p< 0.0001.

The fluorescent dye DiI‐labeled LDL has been widely used to measure the efficiency of LDLR‐dependent cholesterol uptake.^[^ ^43^, ^44^ ^]^ To investigate the potential regulatory role of the miR‐19a/LDLR axis in tumoral cholesterol absorption, we first assessed the exogenous DiI‐LDL uptake capacity of glioma cells (**Figure** **6**A). Flow cytometry analysis showed that upregulation of LDLR (pTSB‐LDLR) significantly increased DiI‐LDL uptake compared to control. Conversely, suppressing LDLR via shRNA (Figure S7F, Supporting Information) or introducing an IDH^R132H^ mutation almost abolished cholesterol uptake (Figure 6B, Figure S7G, Supporting Information). These observations were further confirmed by immunostaining (Figure 6C,D). As cholesterol has been shown to promote cell proliferation and migration in various types of cancer cells, and glioma growth is cholesterol‐dependent, we sought to establish the impact of LDLR on these processes. We found that inhibition of LDLR led to marked suppression of proliferation and invasion of glioma cells (Figure 6E,F). Collectively, these results suggest that the miR‐19a/LDLR axis tightly restrains cholesterol uptake and cell proliferation/migration in IDHmt glioma cells.

### 2.7 IDH Mutation‐Potentiated PERK Mediates Cholesterol Transport in Glioma Cells

**Figure 7:**
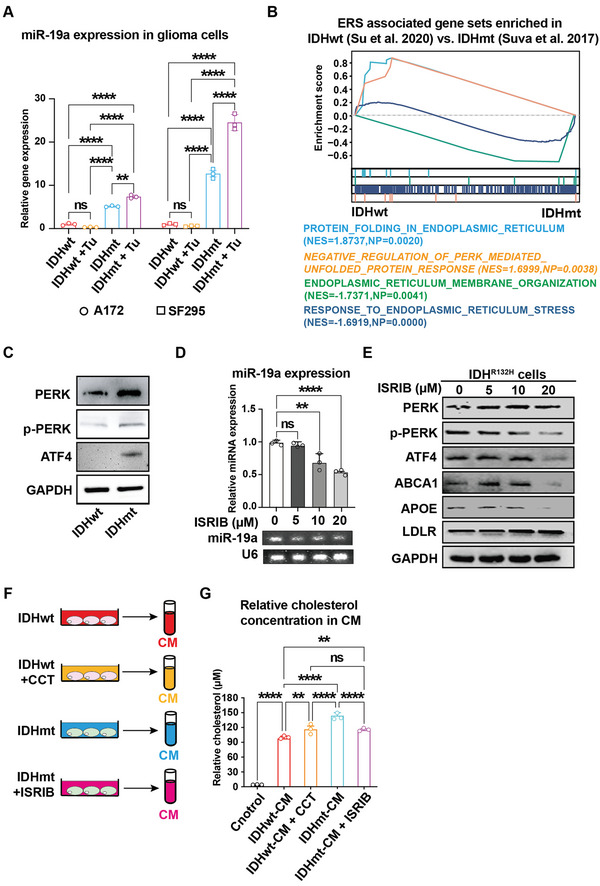
PERK signaling activation results in cholesterol deficits in IDH mutant glioma cells through the miR‐19a/LDLR axis. A) RT‐qPCR analysis of miR‐19a expression levels in different IDH genotypes of A172 and SF295 cells treated with or without 0.5 µg mL^−1^ tunicamycin (Tu) for 24 h (*n* =  3). RNU6B is used as an internal control. B) GSEA of published scRNA‐seq data from clinical samples showing ERS‐associated gene sets and highlighting PERK arm signaling in IDHmt versus IDHwt glioma cells. C) Immunoblotting analysis of PERK, phosphorylated PERK, and ATF4 expression in IDHwt and IDHmt U87 cells. GAPDH is used as a loading control. D) RT‐qPCR analysis of miR‐19a expression in IDHmt U87 cells treated with ISRIB in a concentration gradient for 24 h (*n* =  3). RNU6B is used as an internal control. The RT‐qPCR products are analyzed by gel electrophoresis. E) Immunoblotting analysis of PERK/ATF4 signaling and cholesterol transport‐associated regulators in IDHmt U87 cells treated with a PERK inhibitor, ISRIB, in a concentration gradient. GAPDH is used for a loading control. F) Schematic representation of concentration measurement of extracellular cholesterol released from glioma cells in a PERK‐dependent manner. G) Quantification of extracellular cholesterol concentrations in CM collected from IDHwt U87 cells treated with or without CCT020312 (a PERK inducer) or from IDHmt U87 cells treated with or without ISRIB (a PERK inhibitor) (*n* = 3). Data are shown as means ± SEM. Statistical significance is determined by one‐way ANOVA, ns, *p*> 0.05; **p< 0.01; ****p< 0.0001.

Several key regulatory enzymes involved in cholesterol metabolism are located in the ER, where cholesterol production takes place.^[^ ^45^ ^]^ IDH mutation can disrupt ER homeostasis, leading to the unfolded protein response (UPR) and metabolic changes in glioma cells.^[^ ^32^, ^33^, ^34^ ^]^ Thus, we investigated whether IDH mutation regulates the miR‐19a/LDLR axis via ERS/UPR signaling. As expected, treatment with the ERS inducer tunicamycin (Tu, 0.5 µg mL^−1^) resulted in higher expression levels of miR‐19a, suggesting that it is a signature miRNA of IDHmt cells that exhibits a response to ERS. However, miR‐19a expression remained unchanged in IDHwt cells even with Tu treatment (**Figure** **7**A), reminiscent of the characteristics of IDHwt glioma cells on resistance to ERS, as previously reported.^[^ ^32^ ^]^ To identify which UPR signaling branch induces the miR‐19a/LDLR axis in IDHmt glioma cells, we conducted GSEA using the published scRNA‐seq data and found enriched PERK activation‐mediated UPR signaling in IDHmt glioma cells (Figure 7B). Immunoblotting analysis supported this finding, showing that IDH1 mutation activated the PERK/ATF4 arm of the UPR in U87 glioma cells (Figure 7C), in keeping with our previous report that PERK/ATF4 signals are central ERS sensors that predispose IDHmt cells to apoptosis.^[^ ^32^ ^]^ Remarkably, ISRIB, a potent and selective inhibitor of PERK signaling, blocked miR‐19a expression in IDHmt glioma cells in a dose‐dependent manner (Figure 7D), indicating a regulatory role of the PERK/ATF4 arm in miR‐19a expression.

Further, to determine the involvement of IDHmt in regulating cholesterol transport, specifically influx and efflux, through PERK signaling, we utilized small‐molecule compounds, such as CCT020312 (a PERK agonist) and ISRIB (a PERK antagonist), to modify the cholesterol transport in glioma cells. Remarkably, inhibiting PERK activation via ISRIB concentration gradient resulted in a significant decrease in ATF4 expression, along with a corresponding reduction in ABCA1 and APOE expression, but a steady increase in LDLR expression (Figure 7E), which is in line with the reduction of miR‐19a expression under the same ISRIB treatment. By measuring extracellular cholesterol concentrations, we observed that activating PERK via CCT020312 markedly increased cholesterol efflux from glioma cells into the culture medium, whereas blocking PERK through ISRIB considerably decreased cholesterol secretion (Figure 7F,G). Taken together, our data suggest a possible mechanism by which IDH mutation modulates cholesterol transport via the miR‐19a/LDLR axis and ATF4/ABCA1 transcriptional axis under PERK signaling. These results support our earlier observation that IDHmt cells maintain significantly lower intracellular cholesterol levels compared to IDHwt cells (Figure 3E).

### 2.8 Perturbation of PERK in Gliomas Reprograms Polarization of GAMs via Cholesterol

**Figure 8:**
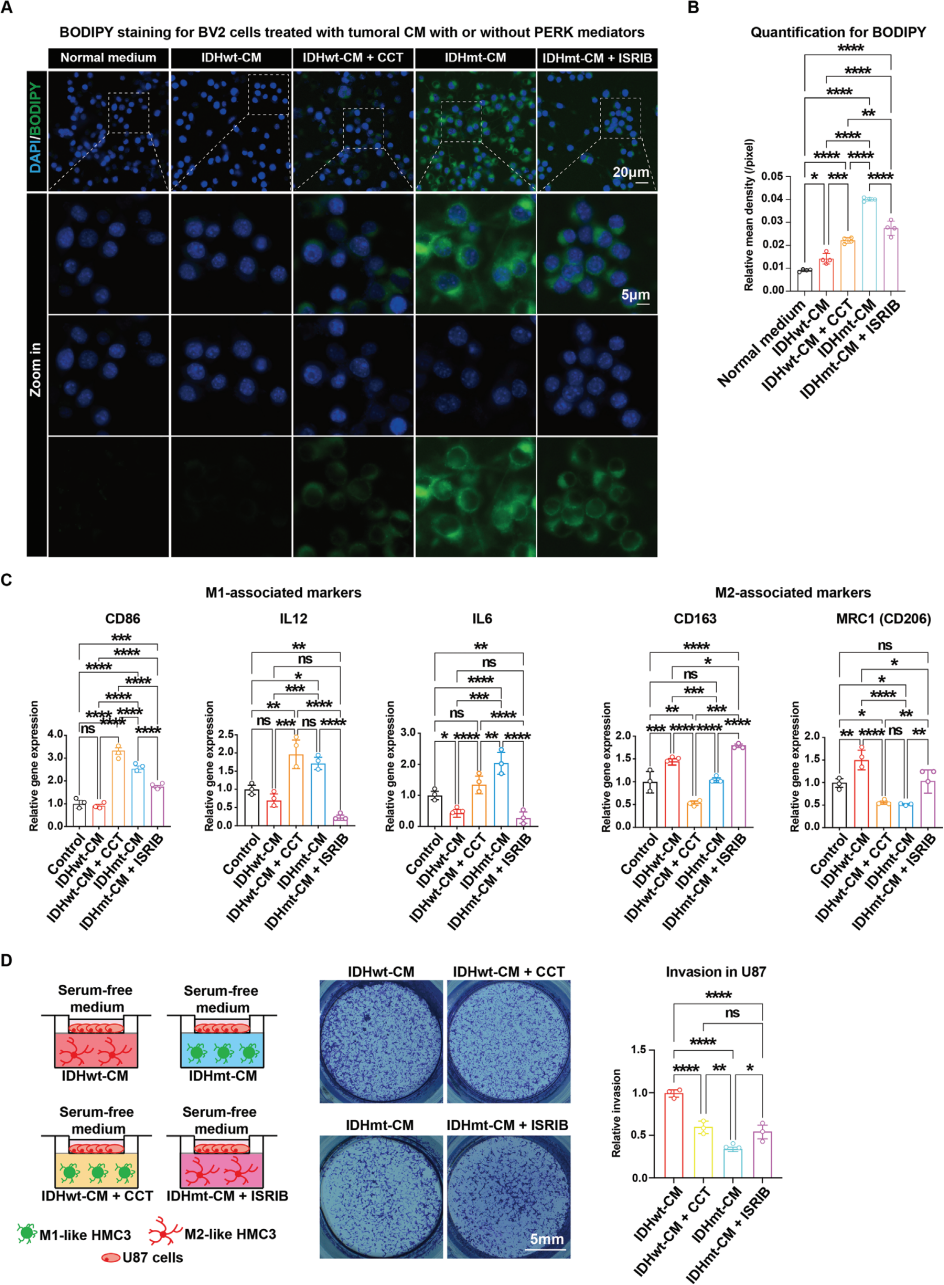
Activated PERK promotes M1 polarization of GAMs, leading to suppression of glioma cell invasion. A,B) Representative images (A) and quantification (B) of BODIPY staining in BV2 cells (*n* =  4) cultured for 48 h in five types of medium: normal DMEM medium, IDHwt‐CM, IDHwt‐CM + CCT, IDHmt‐CM, and IDHmt‐CM + ISRIB. C) RT‐qPCR analysis of microglial polarization‐associated gene expression in HMC3 cells (*n* = 3) cultured for 48 h in the five types of medium as described in (A). ACTB is used as an internal control. D) Schematic representation of the co‐culture system of glioma cells in serum‐free medium and HMC3 microglial cells in CM with or without PERK perturbation (left panel). Representative images (mid panel) and quantification (right panel) of the tumor invasion assay using crystal violet staining in glioma cells co‐cultured with the microglial cells under different conditions (*n* = 3). Data are shown as means ± SEM. Statistical significance is determined by the one‐way ANOVA, ns, *p*> 0.05; *p< 0.05; **p< 0.01; ***p< 0.001; ****p< 0.0001.

We previously reported that IDHmt enhances the ERS sensitivity of glioma cells by activating the PERK/ATF4 axis, suggesting that the PERK arm of UPR signaling could be a potential target for glioma therapy.^[^ ^32^ ^]^ Building on this, we sought to determine whether activated PERK could further trigger the M1‐like polarization of GAMs through promoting cholesterol excretion from glioma cells. To address this question, we tested the cholesterol uptake capacity of BV2 microglial cells and their response to different tumoral CM, with or without the perturbation by PERK mediators (i.e., IDHwt, IDHwt + CCT020312, IDHmt, and IDHmt + ISRIB). BODIPY staining revealed that IDHmt‐CM induced the most significant cholesterol enrichment on the membrane of BV2 cells compared to other treatments. Strikingly, this effect was reversed by ISRIB, resulting in a significant decrease in BODIPY staining intensity in BV2 cells. Conversely, the PERK activator CCT020312, which promotes cholesterol export to the IDHwt‐CM, enhanced the cholesterol uptake of BV2 cells compared to that seen with IDHwt‐CM alone (**Figure** **8**A,B). These findings were consistent with BODIPY staining of HMC3 cells treated with different CM (Figure S8A,B, Supporting Information). RT‐qPCR analysis showed that PERK activation by IDH mutation (IDHmt‐CM) or CCT020312 (IDHwt‐CM + CCT) significantly upregulated M1‐associated gene markers, CD86, IL12, and IL6 (Figure 8C, left panel). In contrast, blocking the PERK signal with archetypal IDH (IDHwt‐CM) or ISRIB (IDHmt + ISRIB) markedly increased the expression of M2‐associated genes, CD163 and MCR1 (Figure 8C, right panel).

To investigate the plausible feedback effect of the PERK/cholesterol‐induced microglial polarization on glioma cell invasion, we established a co‐culture system of glioma and microglia using a transwell assay (Figure 8D, left panel). As expected, HMC3 cells exposed to IDHmt‐CM significantly suppressed the invasion ability of IDHwt U87 cells compared to other treatments. Furthermore, the combination of CCT020312 and IDHwt‐CM also significantly reduced the invasion capacity of glioma cells, similar to the effects of IDHmt‐CM. These findings suggest that PERK activation, induced by IDHmt or synthetic CCT020312, promoted M1‐like microglia, leading to anti‐tumoral effects that inhibited glioma progression. However, this anti‐tumoral effect was attenuated when PERK was inhibited using IDHwt‐CM alone or IDHmt‐CM combined with ISRIB (Figure 8D, middle and right panels). Taken together, these results demonstrate that gliomal PERK mediated‐cholesterol excretion facilitated M1‐like polarization of GAM, which subsequently suppressed glioma progression.

## 3 Discussion

IDH mutations are known as pathologic classifiers and prognostic biomarkers for gliomas, and have also been considered potential therapeutic vulnerabilities. However, targeting IDH mutations in glioma remains challenging due to the involvement of multiple molecular targets and pathways and unclear mechanisms. One alternative approach for glioma therapy is to target the metabolic pathways altered by IDH mutations. In this study, we confirmed the strong correlation between tumoral IDH genotypes and corresponding GAM phenotypes. We determined that IDH genotypes sculpt M1/M2‐like polarization of GAMs using various approaches, including public databases, published datasets, cell lines, mouse models, and clinical samples. Furthermore, we demonstrated that the excess extracellular cholesterol released from IDHmt glioma cells is critical for the pro‐inflammatory (M1‐like) polarization of activated GAMs. Our study also revealed that IDH mutation promotes tumoral cholesterol secretion by reducing cholesterol uptake through the miR‐19a/LDLR post‐transcriptional axis, while enhancing cholesterol efflux via upregulation of ABCA1/APOE, both in a PERK‐dependent manner. Finally, we tested and verified that a synthetic PERK inducer, CCT020312, stimulates cholesterol secretion from glioma cells, which could become a potential targeted drug for glioma from both the tumor and TME perspectives. Collectively, our findings highlight cholesterol as a novel metabolic messenger that modulates GAM phenotypes and influences glioma growth and progression (Figure S9, Supporting Information), thereby providing new mechanistic insights into the functions of cholesterol in glioma biology.^[^ ^1^, ^2^, ^8^ ^]^

Cholesterol plays a critical role in the growth and invasion of glioma, particularly GBM^[^ ^1^, ^2^ ^]^ Studies have shown that glioma cells rely on LDLR‐mediated cholesterol uptake rather than de novo synthesis, and suppression of LDLR expression leads to reduced proliferation, invasion, and migration of glioma cells.^[^ ^1^, ^2^, ^8^ ^]^ Using GL261 cell‐derived syngeneic and lentivirus‐induced spontaneous glioma models, we observed a lower intensity of Filipin staining in IDHmt glioma cells than in IDHwt counterparts, suggesting a cholesterol‐deficit phenotype in IDHmt glioma cells, which is consistent with previous research.^[^ ^8^ ^]^ Yang et al. reported that IDH mutation results in a decline in LDLR protein expression due to elevated expression of LXR and IDOL that facilitates the ubiquitination and degradation of LDLR.^[^ ^8^ ^]^ Our bioinformatics analysis and immunoblotting data did not show significant changes in LXR*β* mRNA and protein expression in the presence or absence of IDH mutation. However, we found significantly reduced LDLR expression in IDHmt clinical samples and cell lines through miR‐19 post‐transcriptional regulation. An array of miRNAs, including miR‐155, miR‐183, and miR‐4476, has been reported as signature miRNAs in IDHmt glioma.^[^ ^32^, ^46^, ^47^ ^]^ In this study, we report a novel IDHmt signature miRNA, miR‐19a, which is up‐regulated in a cellular setting in which IDH mutation or its metabolic product, D2HG, prevails. Furthermore, we found that the expression of miR‐19a could also be promoted by activating the PERK/ATF4 signaling pathway, which subsequently enhances the expression of cholesterol export‐associated molecules, ABCA1 and APOE. Studies have shown that activating PERK/ATF4 promotes cholesterol efflux in hepatocytes by inhibiting the expression of high‐density lipoprotein (HDL) receptor, SR‐BI (scavenger receptor class B, type I)^[^ ^48^ ^]^ and upregulating the transcription levels of ABCA1.^[^ ^35^ ^]^ Thus, targeting the PERK/ATF4 signaling arm of ERS/UPR could be a potential therapeutic approach for glioma from the perspective of cholesterol metabolism.

Glioma, particularly its highest grade, GBM, is known to be an “immunologically cold” tumor with a hyporesponsive and exhausted state of tumor‐infiltrating lymphocyte (TIL).^[^ ^49^, ^50^, ^51^ ^]^ Our analysis of patient samples, consistent with previous reports, showed minimal CD8^+^ T cell infiltration into gliomas, with no significant difference observed between IDHmt and IDHwt gliomas (Figure S10A,B, Supporting Information, IDHmt: IDHwt = 5: 5, *p* = 0.6548). Additionally, we found no significant difference in cholesterol amounts between the two groups (Figure S10C,D, Supporting Information, IDHmt: IDHwt = 5: 5, *p* = 0.9491). Despite their immunologically cold nature, gliomas contain a significant number of GAMs, which play a crucial role in glioma development, and their pro‐inflammatory activation can promote the apoptosis of glioma cells.^[^ ^52^ ^]^ Therefore, GAMs may represent an indispensable and pivotal target for brain tumor immunotherapy. To minimize the effects of variable immune infiltration, we selected only lower‐grade malignant gliomas (IDHmt versus IDHwt) from published databases for subsequent bioinformatic analysis of TME composition, GAM polarization, and expression profiles of genes involved in cholesterol metabolism.

Previous publications have shown that glioma cells can interact with and educate resident or infiltrated GAMs, leading to the transformation of these cells into pro‐tumorigenic phenotypes through the chemotactic interactions between cytokines and receptors, such as C‐X3‐C motif chemokine ligand 1 (CX3CL1)/C‐X3‐C motif chemokine receptor 1(CX3CR1), C‐C motif chemokine ligand 2 (CCL2)/C‐C motif chemokine receptor 2 (CCR2), colony‐stimulating factor 1 (CSF1)/colony stimulating factor 1 receptor (CSF1R).^[^ ^40^, ^41^ ^]^ In addition to cytokines, glioma cell‐derived metabolites, such as 6‐formylindolo [3,2‐b]carbazole (FICZ), kynurenine (Kyn), and 2‐(1′H‐indole‐3′‐carbonyl)‐thiazole‐4‐carboxylic acid methyl ester (ITE), may also contribute to shaping the phenotypes of GAMs.^[^ ^53^, ^54^, ^55^ ^]^ While the role of cholesterol in regulating tumor‐associated macrophages (TAMs) has been reported in other cancers and diseases, its impact on GAM polarization in glioma remains unclear to date. Studies by Goossens et al. and Hoppstädter et al. have shown that ovarian and lung cancer cells induce M2‐like polarization of TAMs by sequestering their cholesterol, resulting in the activation of IL4/PI3K/STAT6 signaling axis in TAMs.^[^ ^9^, ^10^ ^]^ Our study demonstrated that cholesterol, as a metabolite, plays a regulatory role in remodeling GAM polarization. Our BODIPY staining showed that high cholesterol uptake by IDHwt glioma leads to the depletion of membrane cholesterol of BV2 microglial cells, resulting in an M2‐like polarization. Conversely, cholesterol‐enriched conditioned media collected from IDHmt glioma (IDHmt‐CM), or cholesterol alone, can promote M1‐like polarization of HMC3 and BV2 microglial cells. Notably, this pro‐inflammatory polarization induced by excess exogenous cholesterol has not been previously reported in glioma but has been widely reported in neurodegenerative diseases, where cholesterol accumulation contributes to the pro‐inflammatory status of microglia cells.^[^ ^56^, ^57^ ^]^

Dendritic cells (DCs), which originate from bone marrow progenitors, are essential for presenting antigens and inducing activation and differentiation of naïve T lymphocytes.^[^ ^58^, ^59^, ^60^ ^]^ DCs that infiltrate glioma are relatively abundant and share the same cellular origin, monocytes, as GAMs. Our immunofluorescence staining did not reveal any significant differences in the numbers of CD11c^+^ DCs between IDHmt and IDHwt gliomas (Figure S10A,B, Supporting Information, IDHmt: IDHwt = 5: 5, *p* = 0.9739). However, we found that the infiltrated CD11c^+^ DCs in IDHmt murine gliomas contained relatively higher cholesterol content (Filipin^+^) than those in IDHwt counterparts (Figure S10E,F, Supporting Information, IDHmt: IDHwt = 6: 6, *p* = 0.0492). To address whether and how cholesterol possibly affects the biological functions of DCs, we conducted bioinformatic analysis using the published scRNA‐seq datasets and found that DCs in IDHmt glioma highly express MHC Class II Human Leukocyte Antigen (HLA‐DRB5 and HLA‐DRB6), Toll‐like receptors (i.e., TLR3, TLR4, and TLR5, Figure S9G–I, Supporting Information), anti‐tumoral cytokines (i.e., IL1A, IL1B, and TNFA), granulocyte macrophage‐colony stimulating factor 2 receptor (CSF2RA), and NOD‐like receptor thermal protein domain associated protein 3 (NLRP3, for inflammasome activation) (Figure S10G,H, Supporting Information). Immunofluorescence staining on HLA‐DR partially confirmed our findings (Figure S10I,J, Supporting Information, IDHmt: IDHwt = 5: 5, *p* = 0.0349). In keeping with our results, Westerterp et al. reported that cholesterol accumulation activated inflammasome formation in DCs, and the pro‐inflammatory cytokines produced from this process, in turn, enhanced T‐cell activation in a lupus erythematosus (SLE) murine model.^[^ ^61^ ^]^ We, therefore, postulate that excess cholesterol released from IDHmt glioma may accumulate in phagocytic cells, such as GAMs and DCs, and may even be involved in the functional regulations of these cells. However, further investigation is required to determine the explicit correlation between cholesterol accumulation and the pro‐inflammatory activation of these glioma‐associated phagocytes, as well as the underlying regulatory mechanism.

Are there any indirect effects of membrane cholesterol alteration on other membrane lipids that might subsequently contribute to what we observed? To our knowledge, although it is technically challenging to exclude such potential, cholesterol is minimally involved in the metabolism of other major membrane lipids such as phospholipids and triglycerides. In addition to cholesterol transport, excessive cholesterol may be converted into cholesterol esters by sterol O‐acyltransferases (SOAT1 and SOAT2) and stored intracellularly as lipid droplets.^[^ ^62^ ^]^ However, we found no significant difference in the expression of *SOAT1/2* between IDHmt‐CM‐ and IDHwt‐CM‐treated GAMs, as shown in Figure S4, Supporting Information. Additionally, we examined the expression of PLIN2, a constitutive lipid droplet protein, through transcriptomic analysis using in‐house bulk RNA‐seq and the published scRNA‐seq datasets,^[^ ^15^ ^]^ and found no significant difference in PLIN2 expression between IDHmt‐CM‐ and IDHwt‐CM‐treated HMC3 cells (Figure S11A, Supporting Information), which was confirmed by immunofluorescence staining (Figure S11B,C, Supporting Information, IDHmt: IDHwt = 5: 5, *p* = 0.8222). Furthermore, accumulated cholesterol on the cell membrane can bind to sphingolipids and glycolipids to form lipid rafts, which are functional membrane microdomains that organize signal transduction.^[^ ^63^ ^]^ Using the same datasets, we examined the expression of FLOT1, a ubiquitously expressed, conserved lipid raft‐associated scaffolding protein, and found no significant difference in FLOT1 expression between IDHmt‐CM‐ and IDHwt‐CM‐treated HMC3 cells (Figure S11D, Supporting Information), which was confirmed by immunofluorescence staining (Figure S11E,F, Supporting Information, IDHmt: IDHwt = 5: 5, *p* = 0.6798). Taken together, we did not observe significant expression changes of the lipid‐associated proteins PLIN2 and FLOT1 in HMC3 cells in the context of tumoral IDH genotypes. While this evidence is not conclusive, it suggests that it is unlikely that membrane lipids are affected by cholesterol alteration or involved in regulating the polarization of GAMs.

From a clinical standpoint, it is important to determine whether elevated cholesterol levels in peripheral blood are associated with the occurrence and/or progression of glioma. Using our in‐house follow‐up data on 577 patients with glioma (IDHmt: IDHwt = 241: 336), a clinical cohort analysis revealed no significant differences in cholesterol‐associated indices in peripheral blood tests between the two IDH genotypes of gliomas, including LDL‐cholesterol (LDL‐c), triglycerides (TG), HDL‐cholesterol (HDL‐c), apolipoprotein A1 (APOA1), apolipoprotein B (APOB), and total cholesterol (CHO) (Figure S12A, Supporting Information, IDHmt: IDHwt = 241: 336, *p*>0.05). Additionally, our Kaplan‐Meier analysis of overall survival indicated no significant association between hypercholesterolemia (CHO> 5.72 mmol L^−1^; TG< 1.70 mmol L^−1^) or hyperlipidemia (CHO> 5.72 mmol L^−1^; TG> 1.70 mmol L^−1^) and the prognosis of glioma patients (Figure S12B,C, Supporting Information, *p*> 0.8). Consistent with our findings, Cote et al. also reported a similar insignificant correlation of hyperlipidemia with patient prognosis (multivariable HR =  0.96, 95%CI = 0.75–1.25).^[^ ^64^ ^]^

Regarding potential cholesterol‐targeting therapeutic drugs for glioma patients, a comprehensive analysis of the results of 51 pre‐clinical studies worldwide showed that statins could inhibit the proliferation, migration, and invasion of glioma cells, exerting anti‐glioma effects.^[^ ^65^ ^]^ Consistent with this, three anti‐glioma compounds/medicines targeting cholesterol transport have been reported to date, including LXR‐623,^[^ ^1^ ^]^ Efavirenz,^[^ ^2^ ^]^ and Atorvastatin.^[^ ^8^ ^]^ These compounds can penetrate the blood‐brain barrier and efficiently target cholesterol dysregulation in the glioma parenchyma. LXR‐623, as an LXR agonist, reduces the intracellular cholesterol amount of glioma cells. Efavirenz activates the CYP46A1 expression, enhancing 24OHC production, which promotes cholesterol efflux. Atorvastatin, an HMGCR inhibitor, blocks cholesterol biosynthesis. However, six observational studies worldwide found an insignificant inverse correlation between statin use and glioma incidence (HR = 0.84, 95%CI = 0.62–1.13).^[^ ^65^ ^]^ In contrast, Cote et al. reported that glioma patients who used statins for a longer duration had significantly worse survival than those who had never used statins (HR =  1.48, 95%CI = 1.08–1.82 comparing > 8 years of use to never use, *p*‐trend =  0.01).^[^ ^64^ ^]^ Although it appears that the clinical effects of statins on glioma incidence and progression remain inconclusive, further investigation is still required, particularly regarding the newly identified compounds.

In conclusion, our results demonstrate that IDHmt glioma cells secrete excessive cholesterol, which functions as a metabolic messenger, determining the pro‐inflammatory polarization of GAMs. Our findings elucidate the molecular mechanism by which IDHmt‐induced PERK activation alters tumoral cholesterol homeostasis through the miR‐19a/LDLR axis and upregulation of ABCA1/APOE, leading to enhanced cholesterol excretion. Our study underscores the potential of PERK as a therapeutic target for glioma treatment, impacting both the tumor parenchyma and GAMs.

## 4 Experimental Section

### Cell Culture and Transfection

Three human glioma cell lines (A172, RRID: CVCL_0131; SF295, RRID: CVCL_1690; and U87, RRID: CVCL_3428), a murine glioma cell line (GL261, RRID: CVCL_Y003), a human microglial cell line (HMC3, RRID: CVCL_II76), and a murine microglial cell line (BV2, RRID: CVCL_0182) were cultured in Dulbecco's modified Eagle's medium (DMEM; Invitrogen, # 12430054) supplemented with 10% (v/v) FBS (Biological Industries).

Glioma cells stably expressing IDH1^R132H^ (IDHmt) were generated by lentiviral transduction followed by puromycin selection at a final concentration of 1 µg mL^−1^. The lentiviral particles constructed with IDHmt, or corresponding control vector, were purchased from Genechem (genechem.com.cn). For transient transfection, plasmids encoding miR‐19a mimics/antagomirs, pTSB‐LDLR or shLDLR, or corresponding negative controls, were transfected into cells using Lipofectamine 3000 reagent (Invitrogen, #L3000008) following the manufacturer's instructions. The plasmid details are shown in Table S3, Supporting Information.

### D2HG Quantification

The cellular level of D2HG was assessed using an enzymatic assay described previously.^[^ ^32^ ^]^ Briefly, the D2HG detection Kit (Biovison, #K213‐100) was used to determine D2HG levels, and a standard curve was generated using commercial D2HG (0.5, 1, 2.5, 5, 7.5, 10, 25, 50, and 100 µm). To perform the assay, freshly prepared reaction mixes containing D2HG Assay Buffer, D2HG Enzyme, D2HG Substrate Mix, and cell lysates were incubated for 60 min at 37 °C. The optical density was subsequently measured at 450 nm using a Synergy H1 microplate reader (BioTek, USA).

### Conditioned Media (CM) from U87 Glioma Cells

IDHwt and IDHmt U87 cells were seeded in equal numbers per well in 6‐well plates and maintained with the normal culture medium for 48 h. Then, IDHwt U87 cells were cultured with DMEM and treated with or without 2 µm CCT020312 (PERK inducer, MCE, #HY‐119240), while IDHmt U87 cells were cultured in DMEM and treated with or without 10 µm ISRIB (PERK inhibitor, MCE, #HY‐12495) for 48 h. Finally, the cell supernatants were collected, centrifuged at 500xg for 15 min, and filtered through a 0.45 µm filter (Merck, #CLS431220). The filtered CM was stored at −80 °C.

### In Vitro Assays for Cell Proliferation, Invasion, Migration, and Colony Formation

To measure cell viability, cells with control vector, IDH1^R132H^ or shLDLR were respectively seeded at a density of 5 × 10^3^ cells mL^−1^ in a volume of 200 µL per well in 96‐well plates. After 48 or 96 h, 10 µL of CCK‐8 (Beyotime, #C0038) was added to each well. After an additional 2‐h incubation at 37 °C, the absorbance was measured at 450 nm using a Synergy H1 microplate reader (BioTek, USA).

For invasion assays, a 24‐well Transwell chamber (polycarbonate membrane with 8‐µm pore size, Corning, #3422) was used. The filters were pre‐coated with 100 µL ice‐cold 10% Matrigel in cold DMEM on the upper surface. Equal numbers of IDHwt or IDHmt glioma cells were suspended in a serum‐free medium and seeded into the upper compartment of the chamber, while the lower compartment contained a culture medium supplemented with 10% serum. After 24 h, cells were fixed and stained with 0.1% (w/v) crystal violet. The average number of invasive cells on the lower surface of the filter was determined in three randomly chosen visual fields using an inverted light microscope (Olympus, Japan). Experiments were independently repeated three times.

For wound‐healing assays, IDHwt and IDHmt glioma cells were seeded in 6‐well plates and cultured until they reached 80% – 90% confluency. Scratch wounds were created using a 20 µL pipette tip, and the cells were then incubated with the fresh culture medium. Wound healing was assessed and imaged at 0‐, 12‐, and 24‐h intervals using an inverted light microscope (Olympus, Japan). Assays were repeated three times for each cell line. The cell migration activity was quantified by measuring the width of the wound area at different time points relative to the original width of the wound.

For colony formation, glioma cells with or without IDHmt were collected and cultured in serum‐free DMEM medium with 0.2% Matrigel and overlaid onto 0.5 mm thick bottom Matrigel in a 6‐well plate (30 cells per well). The gliomal colonies (>50 cells) were counted under a light microscope after culturing in Matrigel for 10–15 days.

### Glioma and Microglia Cell Co‐Culture Experiments

Co‐culture experiments were conducted using 24‐well transwell inserts with 8‐µm pore size. IDHwt or IDHmt U87 glioma cells were seeded in serum‐free medium on the Matrigel‐coated upper surface of the filters, while HMC3 microglial cells were placed in the lower chamber with tumoral CM. After 24 h of incubation, the cells were fixed and stained with 0.1% (w/v) crystal violet. The average number of invasive cells on the lower surface was determined in three randomly chosen visual fields under an inverted light microscope (Olympus, Japan). Experiments were independently repeated three times. The invasive cell population was measured using ImageJ, and graphs were generated using GraphPad Prism software.

### Reverse Transcription‐Quantitative Polymerase Chain Reaction (RT‐qPCR)

Total mRNA was extracted from glioma and microglial cells using TRIzol reagent (ThermoFisher, #15596018). One microgram of RNA was reverse transcribed into cDNA using the Evo M‐MLV RT Premix for RT‐qPCR kit (Accurate Biology, #AG11706) following the manufacturer's instructions. The mRNA levels of genes of interest (GOI) were determined by qPCR using an SYBR Green kit (AG, #AG11706). The housekeeping gene GAPDH, was used to normalize GOI outputs for glioma cells, while ACTB was used for microglial cells. Fold changes were calculated using the 2^−ΔΔCt^ method.

To detect miR‐19a expression, 1 µg of RNA was reverse transcribed to cDNA using the All‐in‐One miRNA First‐Strand cDNA Synthesis Kit (IgeneBio, #QP014). Mature miRNAs were assayed by using the miRNA qPCR kit (IgeneBio, #QP010). All samples were normalized to RNU6B, an endogenous control. Primer information is shown in Table S1, Supporting Information.

### Immunoblots

Cell lysates were collected in the cold RIPA buffer (Beyotime, #P0013B) supplemented with protease inhibitors (MCE, #HY‐K0010) and phosphatase inhibitors (Roche, #4906837001). Protein concentrations were measured using the BCA kit (ThermoFisher, #23227). Equal amounts of protein samples were subjected to the 10% (w/v) SDS‐PAGE and transferred onto PVDF membranes (Merck Millipore, #ISEQ00010). After blocking with 5% (w/v) non‐fat milk in Tris Buffered Saline with Tween 20 (TBST) for 1 h at room temperature, the membranes were incubated with primary antibodies (diluted in 5% non‐fat milk in TBST) overnight at 4 °C, The following day, the membranes were incubated with secondary antibodies conjugated with HRP (diluted in 5% non‐fat milk in TBST) for 1 h at room temperature. Chemiluminescence was detected using the ECL Prime Western Blotting Detection Kit (Merck Millipore, #WBKLS0100). The details on the antibodies used in this study are listed in Table S2, Supporting Information.

### Intracellular Cholesterol Quantification

IDHwt and IDHmt glioma cells (5 × 10^5^ cells) were lysed with ice‐cold RIPA buffer (Beyotime, #P0013B) containing protease inhibitors (MCE, #HY‐K0010) and phosphatase inhibitors (Roche, #4906837001) on ice for 30 min. The supernatants were collected, and protein concentrations were quantified using the BCA kit (ThermoFisher, #23227). The total cholesterol level was measured with the Tissue Total Cholesterol Assay (TTCA) Kit (Applygen, #E1005) using an equal amount of lysate from each group. All experiments were performed following the manufacturer's instructions.

### Cholesterol Quantification in CM

Based on Section 4.3, different types of CM were collected for cholesterol concentration assay using a TTCA Kit (Applygen, #E1005) and following the manufacturer's instructions.

### Cholesterol Uptake Assay for Imaging or Flow Cytometry

A DiI‐LDL assay was utilized to assess the performance of glioma cells in LDL cholesterol uptake. First, 5 × 10^5^ cells were seeded on glass slides and incubated with DMEM medium supplemented with 10% FBS overnight. The following day, the cells were washed with PBS three times and incubated in serum‐free DMEM medium for 6 h before adding DiI‐LDL (Yiyuan, #YB‐0010) at a final concentration of 25 µg mL^−1^. After an additional 6 h of incubation with DiI‐LDL, the cells were fixed and stained with DAPI (ThermoFisher, #62248). Confocal scanning laser microscopy (LSM 880, Zeiss) with LSM software (ZEN 2011, Zeiss) was used to examine six random fields per sample immediately. Images were acquired at a magnification of 400X using a 10X eyepiece with a 40X objective lens. An area was then selected using the Optical Zoom Function (Zoom = 3) without changing the objective lens, and scanned at the same magnification but with higher resolution.

To prepare cells for flow cytometry, U87 cells expressing IDHwt or shLDLR were seeded at a density of 5 × 10^5^ cells per well in a 24‐well plate and incubated with 25 µg mL^−1^ of DiI‐LDL in DMEM for 6 h. Cells were then dissociated with TrypLE into a single‐cell suspension and analyzed by a flow cytometer (Cytoflex LX, Beckman). All the experiments were repeated three times.

### Luciferase Reporter Gene Assay

Luciferase reporter gene assays were conducted using the Dual‐Luciferase Reporter Assay System (Promega, #E1910) according to the manufacturer's instructions. Briefly, glioma cells were seeded at a density of 1 × 10^5^ cells per well in a 24‐well plate and incubated for 24 h prior to co‐transfection of the pGL3 construct containing wild‐type, mutant, or negative control sequence of LDLR 3’UTR, with the pRL‐TK plasmid containing the Renilla luciferase gene, using Lipofectamine 3000. The sequence details are provided in Table S3, Supporting Information. After 48 h of incubation, the Dual‐Luciferase Reporter Assay Kit (Uelandy, #F6075) was employed to measure firefly luciferase activity, and Renilla luciferase activity was used for normalization. All the experiments were repeated three times.

### Immunohistochemistry (IHC) and Immunofluorescence (IF) Staining

To prepare brain tissue for histological examination, perfusion fixation was conducted using 4% (w/v) paraformaldehyde (PFA). The mouse brain was then submerged and fixed with 10% buffered formalin (v/v). For IHC, tissues were undergone steps involving tissue processing and paraffin embedding. For IF, tissues were embedded in O.C.T. and stored at −80 °C. The details on the antibodies used in this study are listed in Table S2, Supporting Information.

IHC staining was performed as described previously.^[^ ^32^ ^]^ For histological analysis, tumors were sectioned and stained using antibodies against IDH1^R132H^, CD11b, CD86, CD163, LDLR, and CD8a. Mayer's hematoxylin (Dako) was used for counterstaining. Slides were viewed under a Leica DM4B microscope and scanned with a high‐resolution digital slide scanner (Pannoramic SCANII, 3Dhistech).

For IF staining, fixed cells or frozen tumor sections were blocked in 5% normal serum and then stained with the selected primary antibodies: CD86, CD163, CD11c, HLA‐DR, Perillitin‐2, and Flotillin‐1. After three washes with PBS, the cells or sections were incubated with a secondary antibody coupled to Alexa Fluor 488/Alexa Fluor 547 for 1 h at room temperature. The slides were then rinsed and washed three times with PBS, and mounted with the mounting medium containing DAPI (Abcam, #AB104139). Images were captured using a confocal scanning laser microscope (LSM880) with LSM software.

To visualize cellular structures of GAMs following IF staining on glioma samples, 3D surface volumetric rendering reconstructions of cell morphology was conducted. First, Z‐stacks of images (spacing 0.6 µm) were obtained by randomly selecting mCherry^+^ (glioma cells) and GFP^+^ (GAMs) channels using a Zeiss LSM880 equipped with a 63X oil immersion objective lens. Then, the images were processed by smoothing and background subtraction using ImageJ software. Subsequently, a 3D surface rendering of a single Z‐stack of GFP‐positive (Cx3cr1‐eGFP) cells was created using Imaris software (Bitplane AG, Zurich). The number of GAMs surrounding the glioma and the volume of individual GAMs was calculated and compared between IDHwt and IDHmt. Further, the “Filament” plug‐in function in Imaris was used to render the foot processes of GAMs, and the length and surface area of GFP‐positive extensions were measured. Finally, the Xtension of Filament Sholl Analysis was employed to assess the branching patterns of GAM foot processes by superimposing concentric rings 1 µm apart and quantifying the number of the intersections of bifurcation points intersecting each ring.^[^ ^66^ ^]^

### Filipin Staining

Filipin staining was used to assess non‐esterified cholesterol.^[^ ^67^ ^]^ Frozen brain sections were fixed with 4% PFA, followed by overnight incubation with a combination of 20 µg mL^−1^ of Filipin complex (MCE, #HY‐N6716) and primary antibodies in 10% bovine serum albumin in PBS, and then incubated with secondary antibodies. Brain slices from at least three mice were imaged simultaneously using an LSM880 with LSM software.

To visualize the relative abundance of cholesterol in glioma cells, 3D surface volumetric rendering reconstructions of cholesterol (Filipin^+^) and glioma cells (mCherry^+^ or Nestin^+^) were generated using Imaris software. To identify the intracellular cholesterol in glioma cells, an internalized surface of the co‐localized channel of Filipin^+^ and mCherry^+^ (or Nestin^+^) was created using the “Surface” and “Coloc” functions. To assess the differences in cholesterol levels between IDHwt and IDHmt tumors, the ratio of volumes of co‐localized channels of Filipin^+^/mCherry^+^ or Filipin^+^/Nestin^+^ to the total Filipin^+^ volumes was calculated.

### BODIPY Staining

HMC3 and BV2 microglial cells were seeded onto glass coverslips at a density of 2 × 10^5^ cells and cultured for 24 h. They were then incubated with different types of tumoral‐CM for 48 h. After incubation, the cells were fixed and incubated with 1 µg mL^−1^ BODIPY 493/503 in DMSO (ThermoFisher, #D2191) and DAPI (1:5000, ThermoFisher, #62248) for 10 min. The stained cells were washed and mounted onto microscope slides, and randomly selected visual fields per coverslip were imaged using an LSM880 with LSM software. To obtain a higher resolution, the Optical Zoom Function was used, as described above.

### Bulk RNA‐seq on HMC3 Cells

Total RNA was extracted from HMC3 cells using TRIzol Reagent (ThermoFisher, #15596018) following treatment with normal culture medium, IDHwt tumoral‐CM or IDHmt tumoral‐CM. For library preparation, Kapa mRNAseq Hyper prep kit (Roche) was used, and sequencing was performed on the Illumina NS500 Single‐End 75 bp platform by Aptbiotech (aptbiotech.com). The raw RNA‐seq data were then mapped to the UCSC hg38 genome using STAR v.2.5.3a with default parameters. Gene expression levels, measured as fragments per kilobase of transcript per million mapped reads (FPKM), were quantified using RSEM. Differential gene expression analysis was performed using the Limma package,^[^ ^68^ ^]^ and a change with |log2FC|> 1.5 and P. Adjust < 0.005 was considered significant.

### Analysis of Public Databases and Published Datasets

The level 3 bulk RNA‐seq data of lower‐grade gliomas from TCGA (The Cancer Genome Atlas) and CGGA (Chinese Glioma Genome Atlas) were obtained from the Xena platform (xena.ucsc.edu, data version: 2019‐11‐28) and the CGGA platform (cgga.org.cn, data version: 2017‐09‐08), respectively. From the 702 samples in TCGA, 694 samples were selected by removing those without survival and replication information. From the 1015 samples in CGGA, 623 primary tumor samples were selected by removing GBM and recurrent gliomas, as well as those without survival information. The immune microenvironment scores, miRNA expression profiles, cholesterol metabolism‐associated gene expression, and survival data were analyzed.

For cell type enrichment analysis, the abundance of infiltrated immune cell types in specific genotypes of gliomas was assessed using xCell pre‐calculated scores obtained from the xCell website (xcell.ucsf.edu), which utilized data from public databases (TCGA and CGGA).

Gene set enrichment analysis (GSEA) was conducted using the GSEA tool v3.0 with the MsigDB v6.2 gene sets collections (Hallmarks, C5, and C7) as the input gene sets, and the “classical” method used for calculating enrichment scores.^[^ ^69^ ^]^

The scRNA‐seq datasets of IDH mutant astrocytoma and IDH wild‐type astrocytoma were obtained from Suva et al. 2017^[^ ^13^ ^]^ and Su et al. 2020,^[^ ^37^ ^]^ respectively. Cell type annotations and their respective sample origins were retrieved, and downstream analysis was performed using the Seurat package (v3.1.4)^[^ ^70^ ^]^ in R (version 3.6.2). The expression profiles were normalized, and unwanted variation was removed before reducing data dimensionality. The clustering of expression profiles was performed with a resolution parameter set to 0.6. Clusters containing cells of interest were identified by analyzing the expression of canonical markers for glioma cells and GAMs, respectively. Data were visualized using a 2D uniform manifold approximation and projection (UMAP). Cell‐cell communication was identified using the iTALK package (github.com/Coolgenome/iTALK) by analyzing significantly differentially expressed ligands and receptors (sDE‐LR) and matching the ligand‐receptor pairs as sDE‐LR interactions between IDHwt and IDHmt cell populations based on relevant literature.^[^ ^71^, ^72^, ^73^ ^]^

The scRNA‐seq dataset for the expression of lipid metabolism‐associated genes in GAMs, PLIN2, and FLOT1, was downloaded from https://joycelab.shinyapps.io/braintime/.^[^ ^15^ ^]^

### Ethics Statement, Patients, and Specimens

A total of 100 glioma samples were obtained from the Sun Yat‐sen University (SYSU) Cancer Centre in Guangzhou, Guangdong, China (40 glioma surgical specimens) and Shenzhen Hospital of Southern Medical University (SMU) in Shenzhen, Guangdong, China (a tissue microarray consisting of samples from 60 glioma patients) with the informed consent of the patients according to the guidelines of the 1975 Declaration of Helsinki. The experiment of the patient samples was approved by the Institutional Ethics Committee for Clinical Research and Animal Trials of the SYSU Cancer Center (B2022‐246‐01), the Human Ethics Committees at Shenzhen Hospital of SMU (NYSZYYEC20190006), and the Medical Ethics Committee of the Seventh Affiliated Hospital of SYSU (KY‐2021‐065‐01). Clinical information for the human samples is shown in Table S4, Supporting Information.

### Ethics Statement and Animal Studies

All animal studies were approved by the Institutional Animal Care and Use Committee of SYSU (SYSU‐IACUC‐2021‐000796). The Cx3cr1‐eGFP mice were obtained from Bo Peng at Fudan University (China), and they were on a C57BL/6J substrain background, originally purchased from the Jackson Laboratory (JAX, #005582).

For the intracranial syngeneic models, 5 × 10^5^ mCherry‐labeled GL261 cells carrying either wild‐type or mutant IDH1 were collected and suspended in 3 µL of ice‐cold PBS. Cell suspensions were stereotaxically injected into the frontal cortex of 4‐week‐old Cx3cr1‐eGFP mice [coordinates: anteroposterior (AP) 2 mm, mediolateral (ML) 2 mm, dorsoventral (DV) 3 mm] using a 25‐µL microsyringe (RWD, #79006). Tumors were collected approximately 4 weeks post‐injection.

For the transgenic mouse glioma model,^[^ ^38^ ^]^ glioma was induced with mixed lentivirus preparations containing combinations of pTomo‐Ras‐sip53/Ubi‐firefly‐Luciferase‐IRES virus and Ubi‐IDH1^R132H^‐Cherry‐IRES‐puromycin or control virus. Three microliters of mixed lentivirus preparations were stereotaxically injected into the subventricular zone of 6‐to‐8‐week‐old Cx3cr1‐eGFP mice at a rate of 1000 nL per minute [coordinates: anteroposterior (AP) 0 mm, mediolateral (ML) 1 mm, and dorsoventral (DV) 2.3 mm] using a 10‐µL microsyringe (RWD, #80384). Tumor size was monitored and assessed every 7 days by bioluminescence imaging, and tumors were collected approximately 8 weeks post‐injection. For bioluminescence imaging, mice were anesthetized with isoflurane and injected with D‐luciferin (120 mg kg^−1^, Retro‐orbital injection, ROI), and bioluminescent signals in tumors were recorded by using a Xenogen IVIS imaging system.

### Statistical Analysis

All statistical analyses were performed using GraphPad Prism9.0 (GraphPad Prism Software, USA), unless otherwise specified. The Kaplan–Meier survival analysis was conducted using the log‐rank test. Each experiment was performed at least three times. Data were presented as means ± standard error of the mean (SEM), and the statistical tests used were indicated in the figure legends. *P*‐values less than 0.05 were considered statistically significant and denoted as follows: ns (not significant, *p*> 0.05), * (*p*< 0.05), ** (*p*< 0.01), *** (*p*< 0.001), and **** (*p*< 0.0001).

## Conflict of Interest

The authors declare no conflict of interest.

## Author Contributions

T.W., Y.Z., and Y.F. contributed equally to this work. T.W., Y.Z., Y.F., H.D., X.G., J.C., Y.J., C.L., Z.F., Y.G., X.G., and Z.H. performed the experiments and analyzed the data. C.C., S.B., H.L., K.S., and J.Y. provided technical support. N.L., Y.M., and Y.H. designed the experiments and supervised the project. L.N. and T.W. wrote the manuscript. N.L., T.W., Y.Z., Y.F., C.C., S.B., X.S., D.W., and H.L. revised the manuscript. All authors reviewed the manuscript.

## Supporting information

Supporting Information

Supporting Information

## Data Availability

The data that support the findings of this study are available from the corresponding author upon reasonable request.
