# Supplementary material for: PERK‐Mediated Cholesterol Excretion from IDH Mutant Glioma Determines Anti‐Tumoral Polarization of Microglia
Source: Adv Sci (Weinh). 2023 May 11;10(20):2205949. doi: 10.1002/advs.202205949 (PMC10369258; doi:10.1002/advs.202205949)
Supplement: Supplementary file 1 — Supporting Information [file ADVS-10-2205949-s002.pdf]

ADVANCED SCIENCE
Open Access

Supporting Information

for *Adv. Sci.*, DOI 10.1002/advs.202205949

PERK-Mediated Cholesterol Excretion from IDH Mutant Glioma Determines Anti-Tumoral Polarization of Microglia

Tao Wang, Yunxia Zhou, Yunping Fan, Hao Duan, Xiaoyu Guo, Jinlong Chang, Youheng Jiang, Changxue Li, Zhang Fu, Yunfei Gao, Xiaoran Guo, Kastytis Sidlauskas, Zhenqiang He, Clive Da Costa, Xia Sheng, Dinglan Wu, Jinqiu Yuan, Huiliang Li, Yulong He, Yonggao Mou* and Ningning Li**

Supporting Information

PERK-mediated cholesterol excretion from IDH mutant glioma determines anti-tumoral polarization of microglia

Tao Wang[1, †], *Yunxia Zhou*[1, †], *Yunping Fan*[1, 2, †], *Hao Duan*[3], *Xiaoyu Guo*[3], *Jinlong Chang*[1], *Youheng Jiang*[1], *Changxue Li*[1], *Zhang Fu*[1], *Yunfei Gao*[1, 2], *Xiaoran Guo*[1], *Kastytis Sidlauskas*[4], *Zhenqiang He*[3], *Clive Da Costa*[5], *Sebastian Brandner*[6], *Xia Sheng*[7], *Dinglan Wu*[8], *Jinqiu Yuan*[9], *Huiliang Li*[10,12], *Yulong He*[1, 11, *], *Yonggao Mou*[3, *], *Ningning Li*[1, 12, *]

List of Items in Supporting Information

Figure S1	**Gliomas with different IDH genotypes exhibit distinctive immune infiltration and GAM polarization**
Figure S2	**Coupling of different IDH genotypes with distinct GAM polarization in spontaneous glioma mouse models**
Figure S3	**IDH mutant glioma cell culture phenocopies the biological features of IDH mutant glioma**
Figure S4	**Remodeling HMC3 microglial polarization using glioma cell conditioned medium**
Figure S5	**Remodeling BV2 microglial polarization by cholesterol**
Figure S6	**Differential regulation of cholesterol transport in gliomas with different IDH genotypes**
Figure S7	**Downregulation of LDLR by miR-19a reduces cholesterol influx in glioma cells**
Figure S8	**PERK activation induces M1-like polarization of HMC3 cells**
Figure S9	**Working model illustrating how gliomal IDH genotypes regulate GAM polarization via the miR-19a/LDLR axis-mediated cholesterol export in a PERK-dependent manner**
Figure S10	**Potential impact of cholesterol accumulation in the infiltrated DCs and $CD8^+$ T cells between IDHmt and IDHwt gliomas**
Figure S11	**Expression of lipid-associated molecules, PLIN2 and FLOT1, in GAMs**

Figure S12	**Insignificant correlation of hypercholesterolemia/hyperlipidemia with the prognosis of glioma patients**
Table S1	**The primer sequences used in this study**
Table S2	**The antibodies used in this study**
Table S3	**The vectors and relevant seqeunces used in this study**
Table S4	**The clinical information relevant to this study**

Figure S1

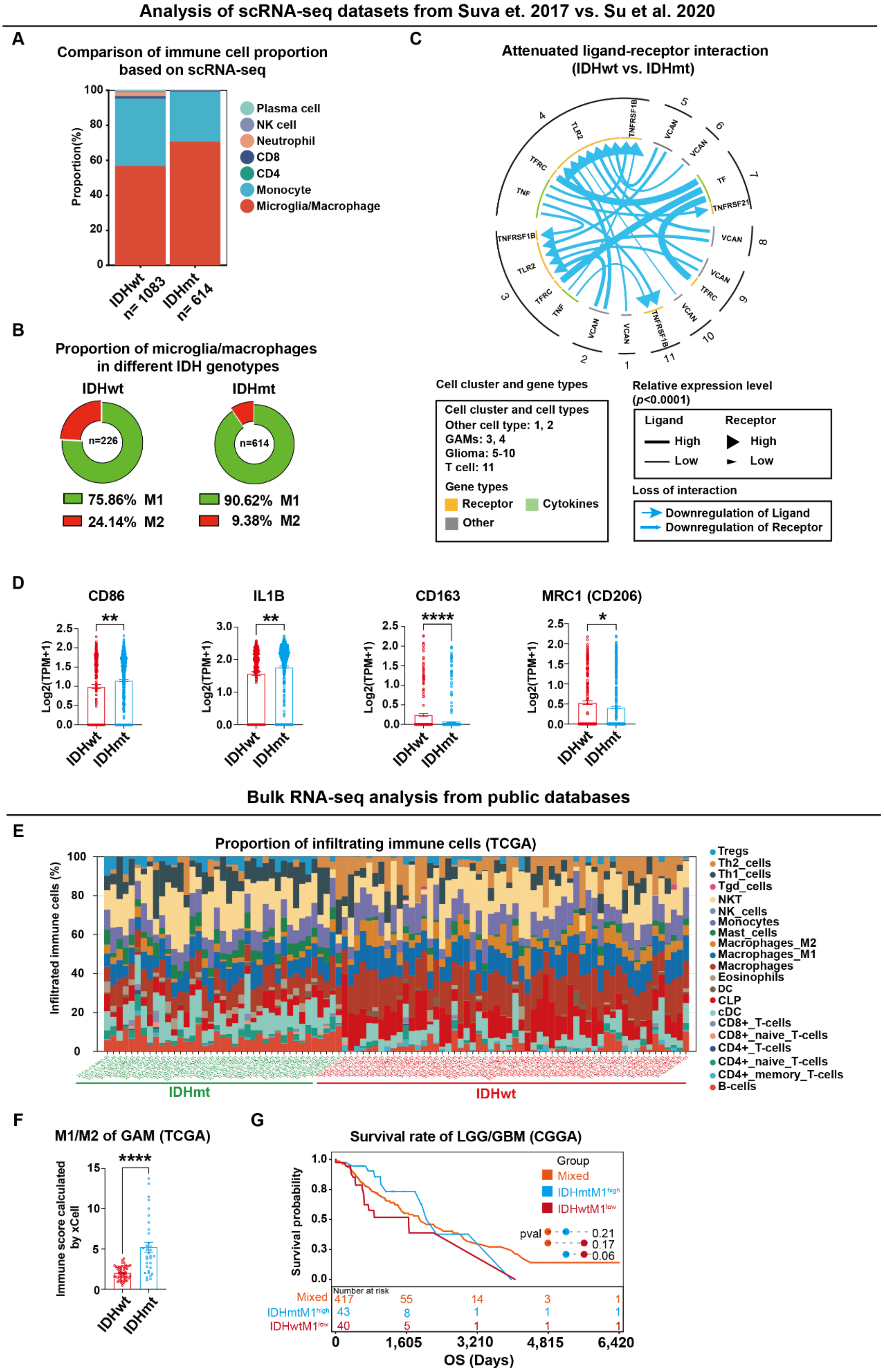

Analysis of scRNA-seq datasets from Suva et. 2017 vs. Su et al. 2020
A
Comparison of immune cell proportion based on scRNA-seq
Proportion(%)
Plasma cell
NK cell
Neutrophil
CD8
CD4
Monocyte
Microglia/Macrophage
IDHwt n= 1083
IDHmt n= 614
B
Proportion of microglia/macrophages in different IDH genotypes
IDHwt
IDHmt
n=226
n=614
75.86% M1
24.14% M2
90.62% M1
9.38% M2
C
Attenuated ligand-receptor interaction (IDHwt vs. IDHmt)
Cell cluster and gene types
Cell cluster and cell types
Other cell type: 1, 2
GAMs: 3, 4
Glioma: 5-10
T cell: 11
Gene types
Receptor
Cytokines
Other
Relative expression level (p<0.0001)
Ligand
Receptor
High
Low
Loss of interaction
Downregulation of Ligand
Downregulation of Receptor
D
CD86
IL1B
CD163
MRC1 (CD206)
Log2(TPM+1)
IDHwt
IDHmt
Bulk RNA-seq analysis from public databases
E
Proportion of infiltrating immune cells (TCGA)
Infiltrated immune cells (%)
Tregs
Th2_cells
Th1_cells
Tgd_cells
NKT
NK_cells
Monocytes
Mast_cells
Macrophages_M2
Macrophages_M1
Macrophages
Eosinophils
DC
CLP
cDC
CD8+_T-cells
CD8+_naive_T-cells
CD4+_T-cells
CD4+_naive_T-cells
CD4+_memory_T-cells
B-cells
IDHmt
IDHwt
F
M1/M2 of GAM (TCGA)
Immune score calculated by xCell
IDHwt
IDHmt
G
Survival rate of LGG/GBM (CGGA)
Survival probability
Group
Mixed
IDHmtM1high
IDHwtM1low
pval
0.21
0.17
0.06
Number at risk
Mixed 417 55 14 3 1
IDHmtM1high 43 8 1 1 1
IDHwtM1low 40 5 1 1 1
0 1,605 3,210 4,815 6,420
OS (Days)

Figure S1. Gliomas with different IDH genotypes exhibit distinctive immune infiltration and GAM polarization. (A) Comparison of immune cell compositions and proportions between IDHmt and IDHwt gliomas (IDHmt: IDHwt= 1,083: 614) using SingleR annotation for published single-cell RNA-seq datasets. **(B)** Pie charts showing the proportion of M1/M2-like GAMs in IDHmt and IDHwt gliomas. **(C)** Circos plot depicting significantly attenuated signaling of ligand-receptor interaction in different cell clusters of IDHwt gliomas relative to that of IDHmt counterparts. Significantly differentially expressed genes (DEGs) are obtained by comparing normalized FPKM counts of relevant scRNA-seq datasets using the Wilcoxon method. Top 20 altered ligand-receptor interactions are selected using the iTALK package built-in ligand-receptor database. Attenuated ligand-receptor interactions are defined as unilateral downregulation of either the ligand (shown in arrow shafts) or receptor (shown in arrowheads), and illustrated by blue arrows depicting the direction of each interaction. **(D)** Expression of polarization-associated genes in GAMs based on scRNA-seq datasets and gliomal IDH classification (IDHmt: IDHwt= 614: 226). **(E)** Proportions of infiltrating immune cells in IDHwt and IDHmt samples from TCGA (IDHmt: IDHwt= 39: 57). **(F)** Comparison of GAM polarization between IDHwt and IDHmt samples from TCGA (IDHmt: IDHwt= 39: 57) using a ratio of M1/M2 calculated by the cell-type enrichment score from xCell. **(G)** Kaplan-Meier curve analysis combining IDH genotypes and M1-like GAM infiltration shows the overall survival of glioma patients carrying IDHmt with high M1-like GAMs, IDHwt with low M1-like GAMs, and mixed classification using CGGA database (IDHmt: IDHwt=

325: 287). The cell-type enrichment score is obtained from xCell using the CGGA database. Samples with the top quartile of M1 macrophage score are defined as high M1 macrophage infiltration (M1^{high}), while samples with the bottom quartile score are defined as low M1 macrophage infiltration (M1^{low}). Data are presented as the means ± SEM. Statistical significance is determined by the two-tailed Student's t-test, *, $p< 0.05$; **, $p< 0.01$; ****, $p< 0.0001$.

Figure S2

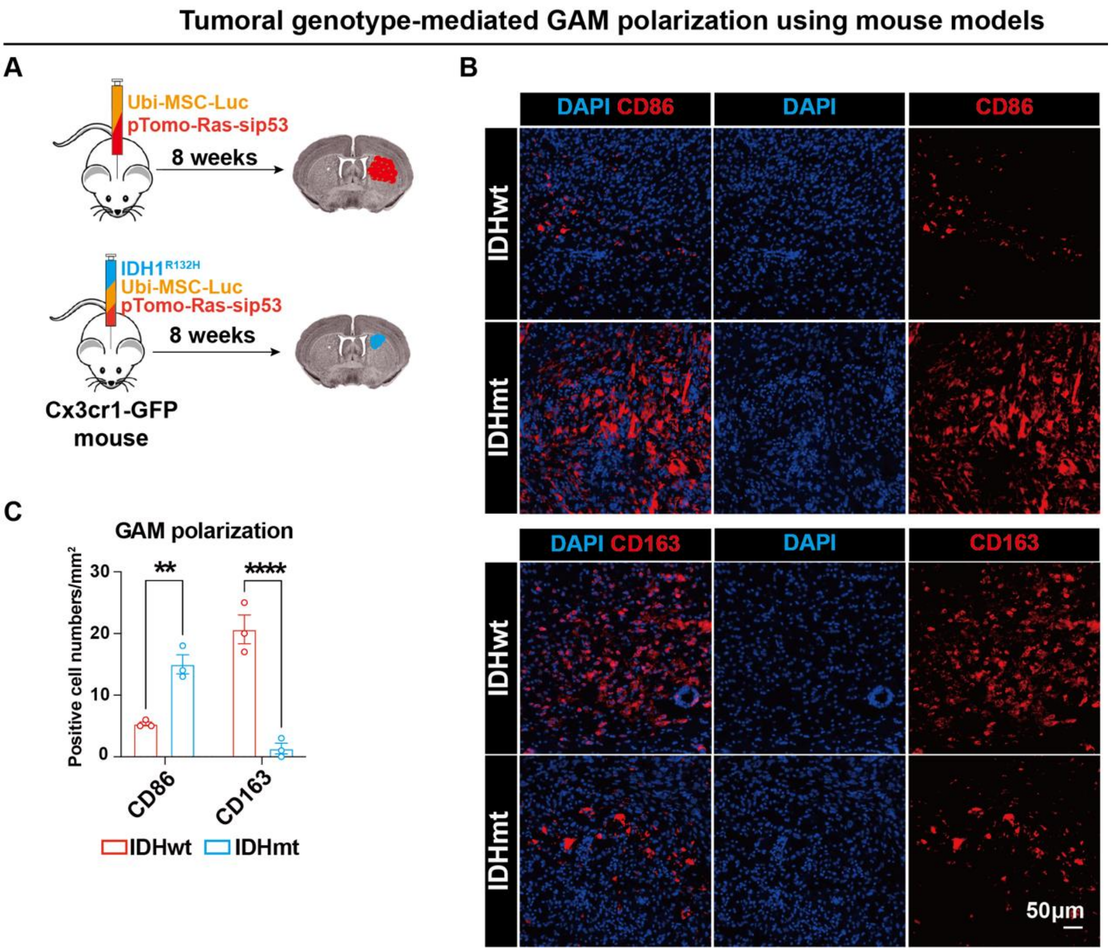

Figure S2. Coupling of different IDH genotypes with distinct GAM polarization in spontaneous glioma mouse models. (A) Schematic representation of the generation of spontaneous glioma mouse models of different IDH genotypes through

stereotaxic injection of lentivirus carrying pTomo-Ras-sip53 and Ubi-MSC-Luc ± IDH^{R132H}. **(B-C)** Representative images (B) and quantification (C) of immunofluorescence staining for CD86 and CD163 in the murine tumors with different IDH genotypes (IDHmt: IDHwt= 3: 3). Data are shown as means ± SEM. Statistical significance is determined by the two-tailed Student's t-test, **, $p< 0.01$; ****, $p< 0.0001$.

Figure S3

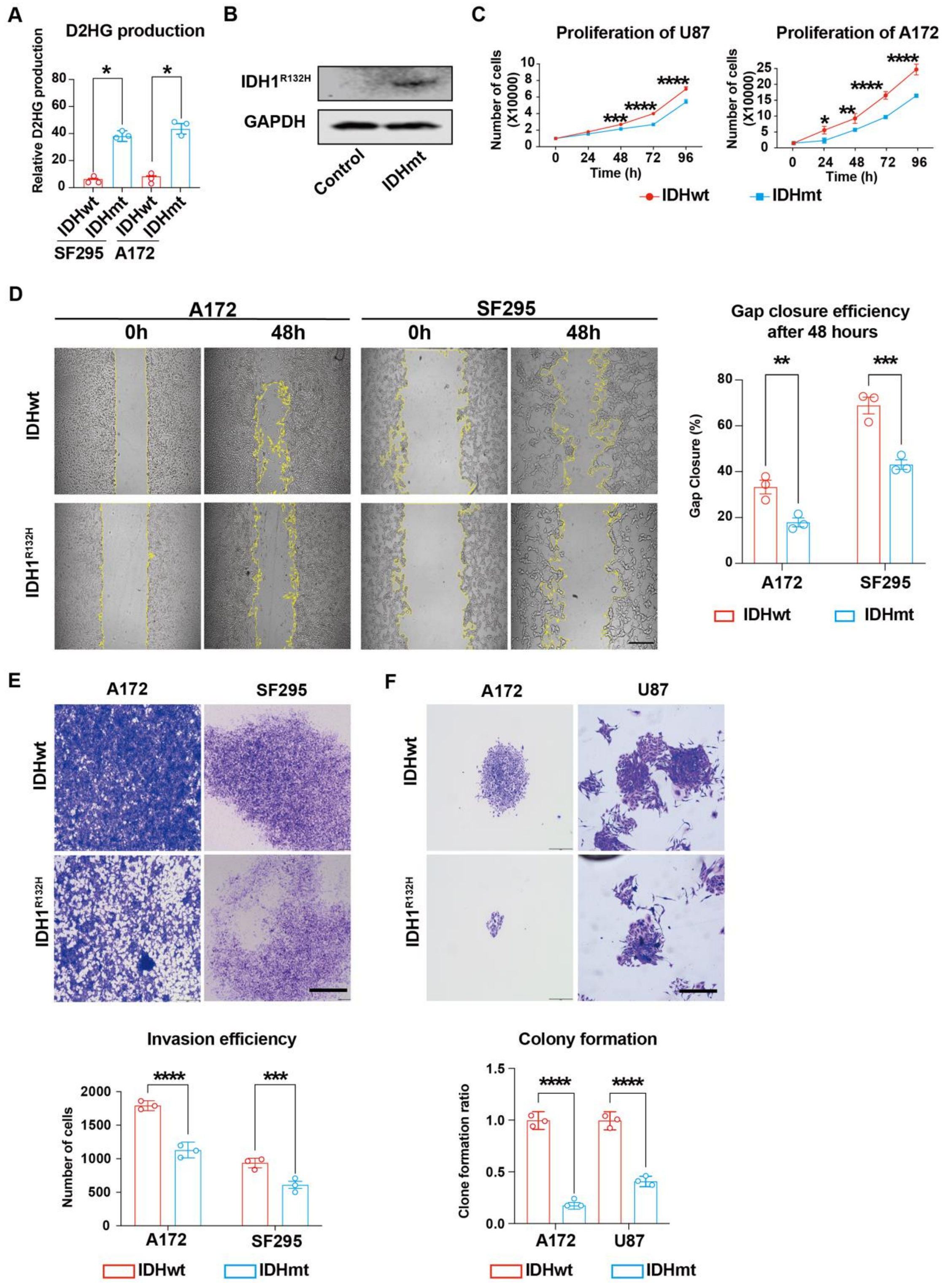

Figure S3. IDH mutant glioma cell culture phenocopies the biological features of IDH mutant glioma. (A) Analysis of intracellular D2HG production in IDHwt vs. IDHmt glioma cells (IDHmt: IDHwt= 3: 3). **(B)** Western blotting analysis confirms

stable expression of exogenous $IDH1^{R132H}$ in A172 cells. GAPDH is used as a loading control. **(C)** The CCK-8 proliferation assay shows the growth curves of glioma cell lines U87 and A172 infected with lenti-control or lenti-$IDH1^{R132H}$ (IDHmt: IDHwt= 3: 3). **(D)** The gap closure assay demonstrates the migratory ability of A172 and SF295 cells infected with lenti-control or lenti-$IDH1^{R132H}$ (IDHmt: IDHwt= 3: 3). **(E)** The transwell assay shows the invasive ability of A172 and SF295 cells infected with lenti-control or lenti-$IDH1^{R132H}$ (IDHmt: IDHwt= 3: 3). **(F)** The colony formation assay illustrates the clonogenic potential of A172 and U87 cells infected with lenti-control or lenti- $IDH1^{R132H}$ (IDHmt: IDHwt= 3: 3). Data are presented as means ± SEM. Statistical significance is determined by the two-tailed Student's t-test, *, $p < 0.05$; **, $p < 0.01$; ***, $p < 0.001$; ****, $p < 0.0001$.

Figure S4

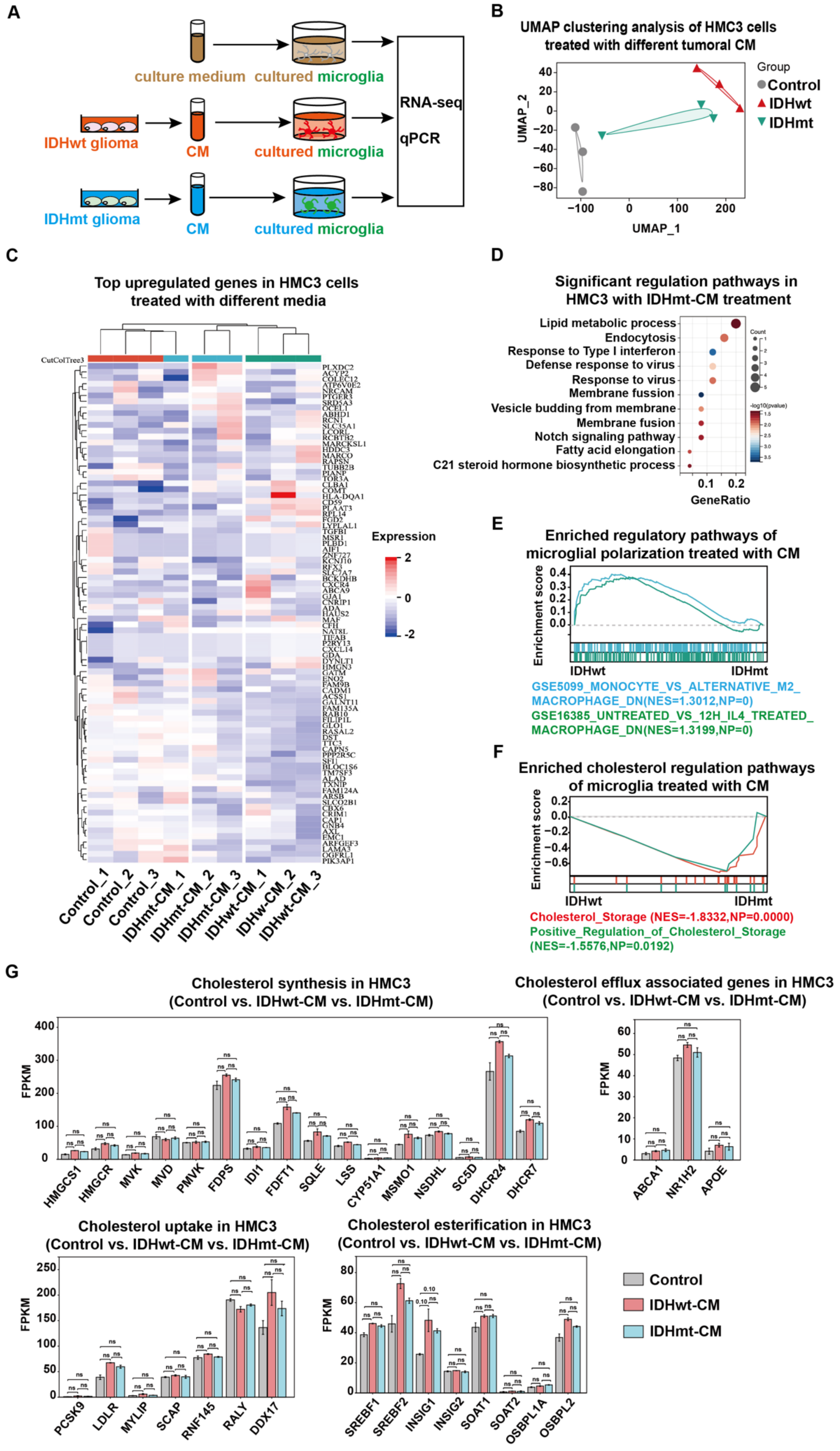
A
culture medium
cultured microglia
IDHwt glioma
CM
cultured microglia
IDHmt glioma
CM
cultured microglia
RNA-seq
qPCR
B
UMAP clustering analysis of HMC3 cells treated with different tumoral CM
UMAP_2
UMAP_1
Group
Control
IDHwt
IDHmt
C
Top upregulated genes in HMC3 cells treated with different media
CutColTree3
Expression
Control_1
Control_2
Control_3
IDHmt-CM_1
IDHmt-CM_2
IDHmt-CM_3
IDHwt-CM_1
IDHw-CM_2
IDHwt-CM_3
D
Significant regulation pathways in HMC3 with IDHmt-CM treatment
Lipid metabolic process
Endocytosis
Response to Type I interferon
Defense response to virus
Response to virus
Membrane fussion
Vesicle budding from membrane
Membrane fusion
Notch signaling pathway
Fatty acid elongation
C21 steroid hormone biosynthetic process
GeneRatio
Count
-log10(pvalue)
E
Enriched regulatory pathways of microglial polarization treated with CM
Enrichment score
IDHwt
IDHmt
GSE5099_MONOCYTE_VS_ALTERNATIVE_M2_MACROPHAGE_DN(NES=1.3012,NP=0)
GSE16385_UNTREATED_VS_12H_IL4_TREATED_MACROPHAGE_DN(NES=1.3199,NP=0)
F
Enriched cholesterol regulation pathways of microglia treated with CM
Enrichment score
IDHwt
IDHmt
Cholesterol_Storage (NES=-1.8332,NP=0.0000)
Positive_Regulation_of_Cholesterol_Storage (NES=-1.5576,NP=0.0192)
G
Cholesterol synthesis in HMC3 (Control vs. IDHwt-CM vs. IDHmt-CM)
FPKM
HMGCS1
HMGCR
MVK
MVD
PMVK
FDPS
IDI1
FDFT1
SQLE
LSS
CYP51A1
MSMO1
NSDHL
SC5D
DHCR24
DHCR7
Cholesterol efflux associated genes in HMC3 (Control vs. IDHwt-CM vs. IDHmt-CM)
FPKM
ABCA1
NR1H2
APOE
Cholesterol uptake in HMC3 (Control vs. IDHwt-CM vs. IDHmt-CM)
FPKM
PCSK9
LDLR
MYLIP
SCAP
RNF145
RALY
DDX17
Cholesterol esterification in HMC3 (Control vs. IDHwt-CM vs. IDHmt-CM)
FPKM
SREBF1
SREBF2
INSIG1
INSIG2
SOAT1
SOAT2
OSBPL1A
OSBPL2
Control
IDHwt-CM
IDHmt-CM

Figure S4. Remodeling HMC3 microglial polarization using glioma cell conditioned medium. (A) Schematic illustration of microglia culture treated with normal growth medium (control) and conditioned medium (CM) collected from IDHwt or IDHmt glioma cells. **(B)** Uniform Manifold Approximation and Projection (UMAP) analysis of RNA-seq depicts three clusters of HMC3 cells treated with control medium, IDHwt-CM, or IDHmt-CM. **(C)** Heatmap displays DEGs from the RNA-seq on HMC3 cells treated with control medium, IDHwt-CM, or IDHmt-CM. Gene expression data are z-transformed for visualization, with red indicating high expression and blue indicating low expression. **(D)** Hallmark enrichment analysis of DEGs from RNA-seq on HMC3 cells treated with IDHmt-CM vs. IDHwt-CM. **(E-F)** Gene Set Enrichment Analysis (GSEA) of RNA-seq on HMC3 cells treated with IDHwt-CM vs. IDHmt-CM, showing enrichment of gene sets associated with polarization regulation (E) and cholesterol storage (F). **(G)** Expression of genes associated with cholesterol synthesis, efflux, uptake, and esterification in HMC3 cells treated with or without tumoral CM (n= 3) based on in-house RNA-seq data. Data are shown as the means ± SEM. Statistical significance is determined by the Kruskal-Wallis test, ns> 0.05.

Figure S5

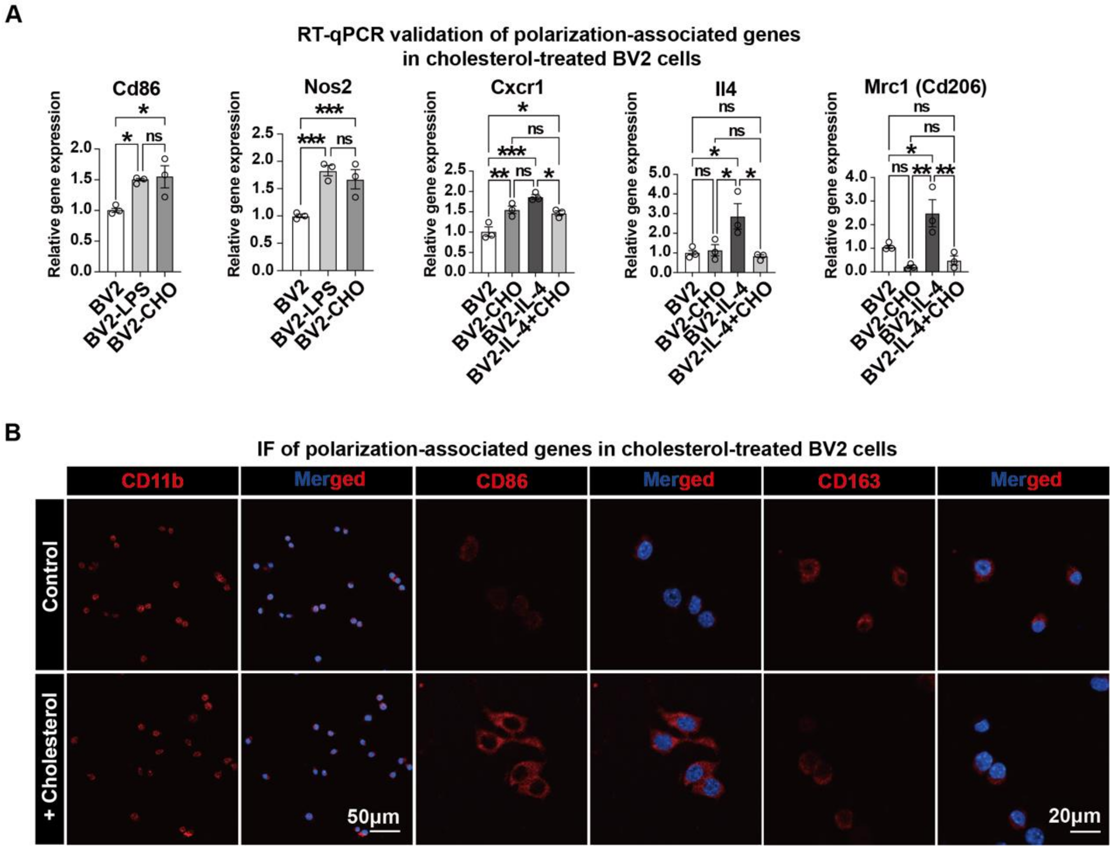

Figure S5. Remodeling BV2 microglial polarization by cholesterol. (A) RT-qPCR analysis of the expression of polarization-associated genes in BV2 cells treated with or without cholesterol (CHO) for 48 hours (n= 3). *Actb* is used as an internal control. LPS and IL-4 are used to induce of M1- or M2-like polarization, respetively. Data are shown as means ± SEM. Statistical significance is determined by one-way ANOVA, ns, p>0.05; **, p< 0.01; ***, p< 0.001; ****, p< 0.0001. **(B)** Representative images of immunofluorescence staining showing the expression of CD86 and CD163 in BV2 microglial cells treated with or without cholesterol.

Figure S6

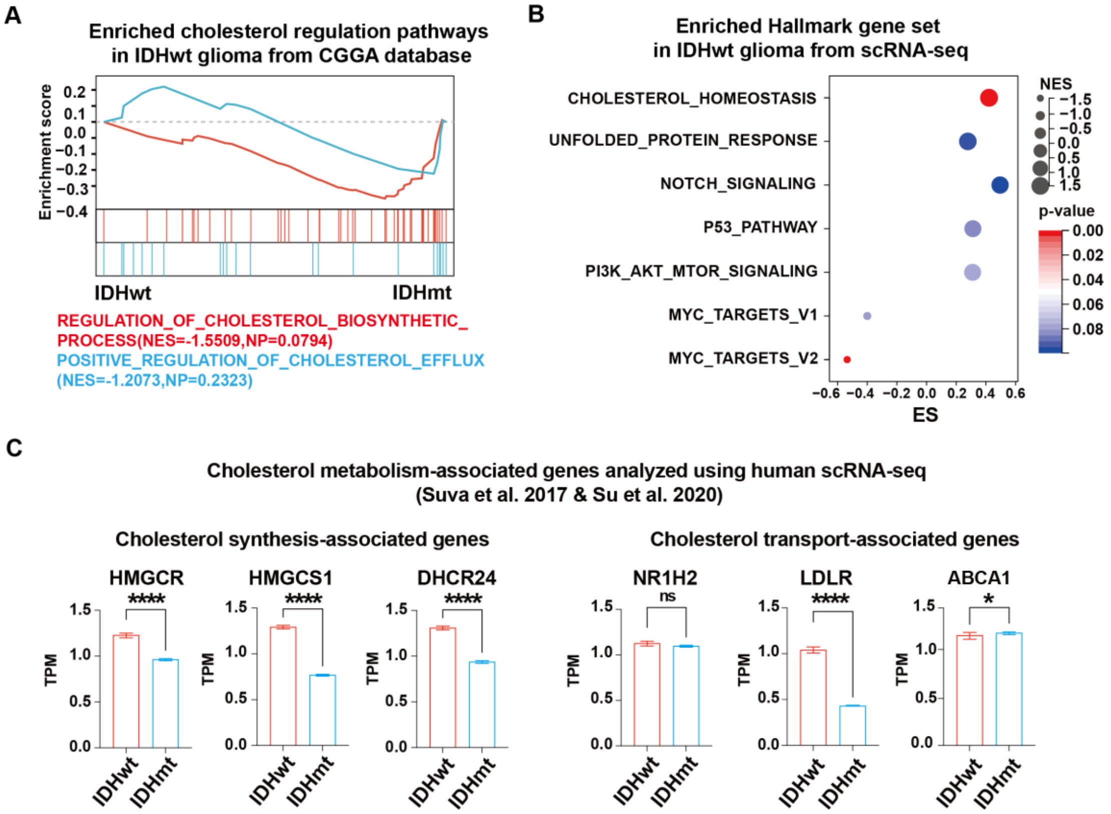

Figure S6. Differential regulation of cholesterol transport in gliomas with different IDH genotypes. (A) GSEA of RNA-seq data from CGGA showing enrichment of cholesterol regulation-related gene sets in IDHmt gliomas. **(B)** Hallmark enrichment analysis of DEGs using published scRNA-seq datasets. **(C)** Expression analysis of DEGs associated with cholesterol synthesis and transport based on published scRNA-seq datasets and IDH classification (IDHmt: IDHwt= 3076: 320). Data are shown as the means ± SEM. Statistical significance is determined by the two-tailed Students' t-test, ns, p>0.05; *, p< 0.05; ****, p< 0.0001.

Figure S7

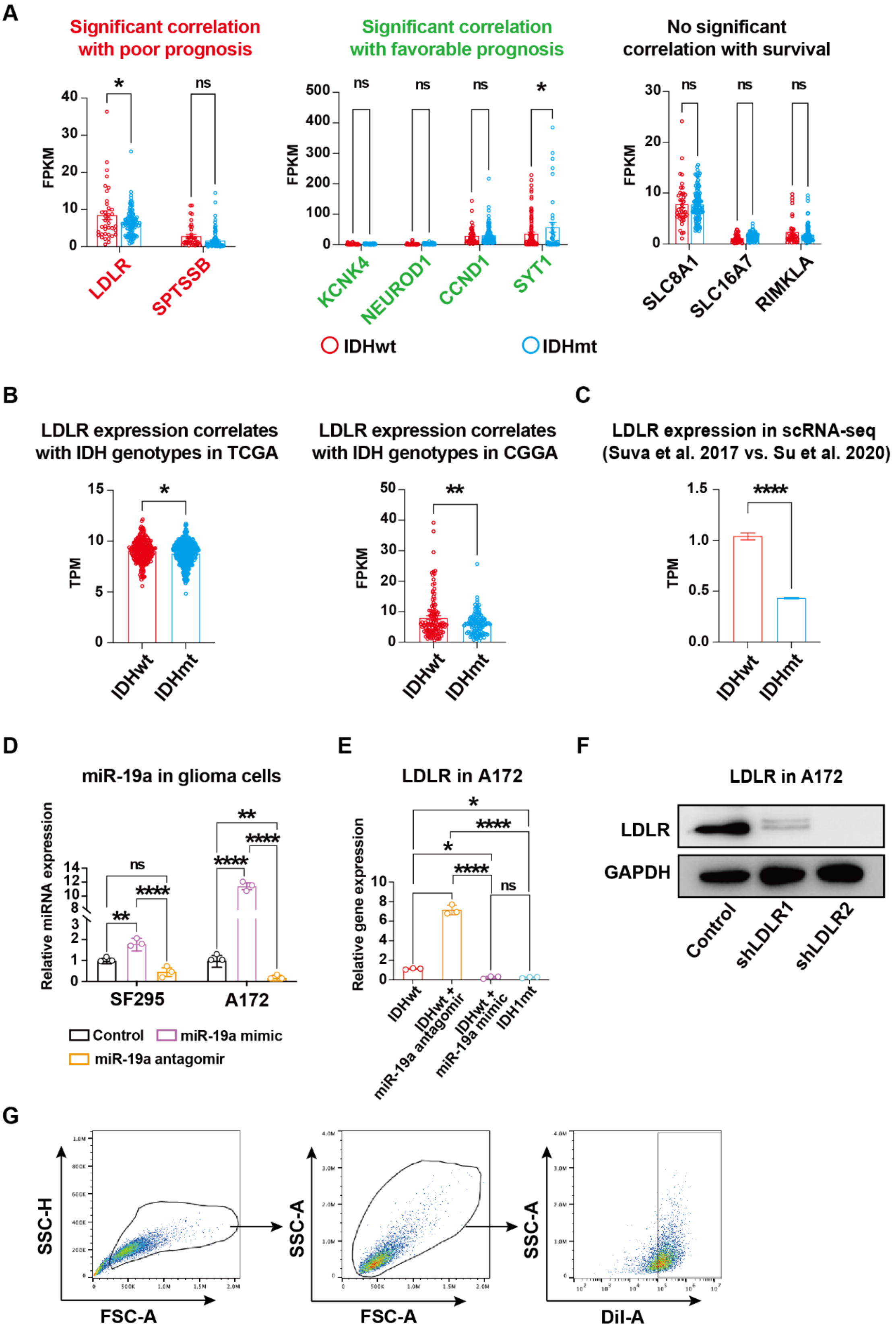

Figure S7. Downregulation of LDLR by miR-19a reduces cholesterol influx in

glioma cells. (A) Expression analysis of miR-19a target genes associated with different prognosis outcomes using CGGA325 datasets based on IDH classification (IDHmt: IDHwt= 38: 105). **(B-C)** LDLR expression analysis from TCGA, CGGA693 (B), and published scRNA-seq (C) datasets based on IDH classification (TCGA, IDHmt: IDHwt= 426: 235; CGGA, IDHmt: IDHwt=209: 176; published scRNA-seq, IDHmt: IDHwt= 987: 256). **(D)** RT-qPCR analysis of miR-19a expression in SF295 and A172 cells transfected with control vector, miR-19a mimics, or antagomirs (*n*= 3). *RNU6B* is used as an internal control. **(E)** RT-qPCR analysis of LDLR expression in A172 cells transfected with control vector, $IDH1^{R132H}$ vector, miR-19a mimics, or antagomirs (*n*= 3). *GAPDH* is used as an internal control. **(F)** Immunoblotting analysis of LDLR expression levels in A172 cells transfected with Lenti-control or Lenti-LDLR shRNA vectors. GAPDH is used as a loading control. **(G)** Selection of a population of single cells using a flow cytometer to detect DiI signal. Data are shown as means ± SEM. Statistical significance is determined by the two-tailed Student's t-test (A-C) or one-way ANOVA (D-E), ns, $p>0.05$; *, $p< 0.05$; **, $p< 0.01$; ****, $p< 0.0001$.

Figure S8

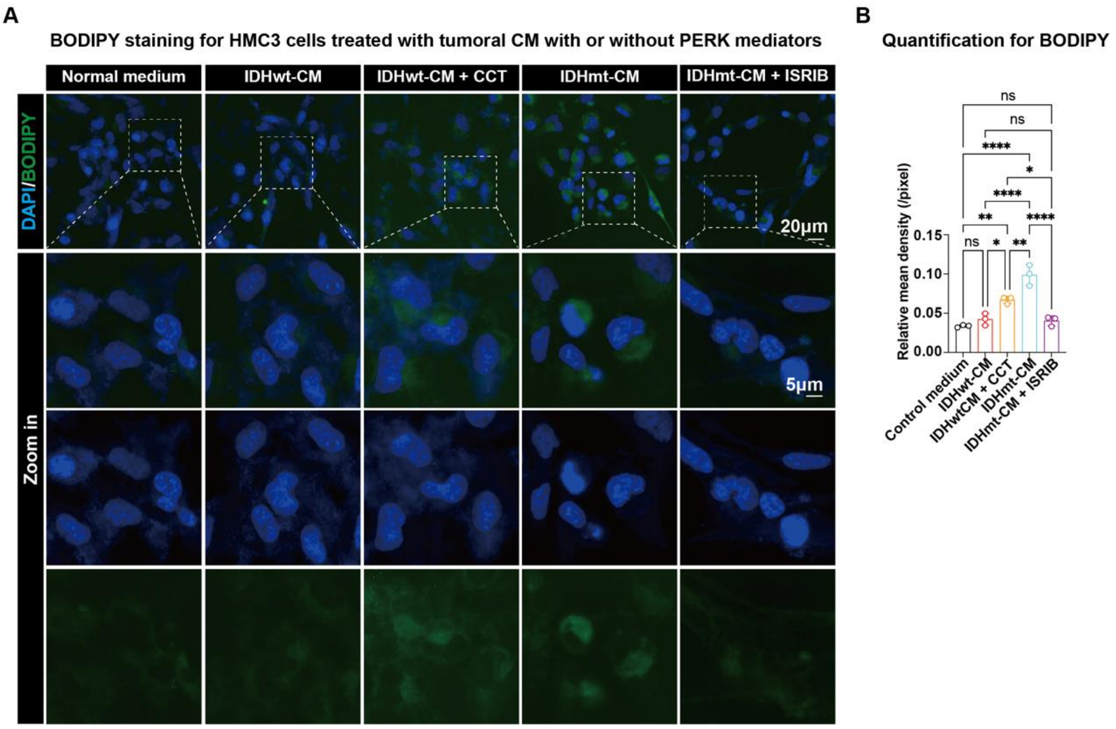

Figure S8. PERK activation induces M1-like polarization of HMC3 cells. (A-B) Representative images (A) and quantification (B) of BODIPY staining in HMC3 cells cultured for 48 hours in control DMEM medium or four types of tumoral CM collected from IDHwt U87 cells treated with or without CCT020312 (a PERK inducer) or from IDHmt U87 cells treated with or without ISRIB (a PERK inhibitor). Data are shown as means ± SEM (n= 3). Statistical significance is determined by the one-way ANOVA, ns, $p > 0.05$; *, $p < 0.05$; **, $p < 0.01$; ****, $p < 0.0001$.

Figure S9

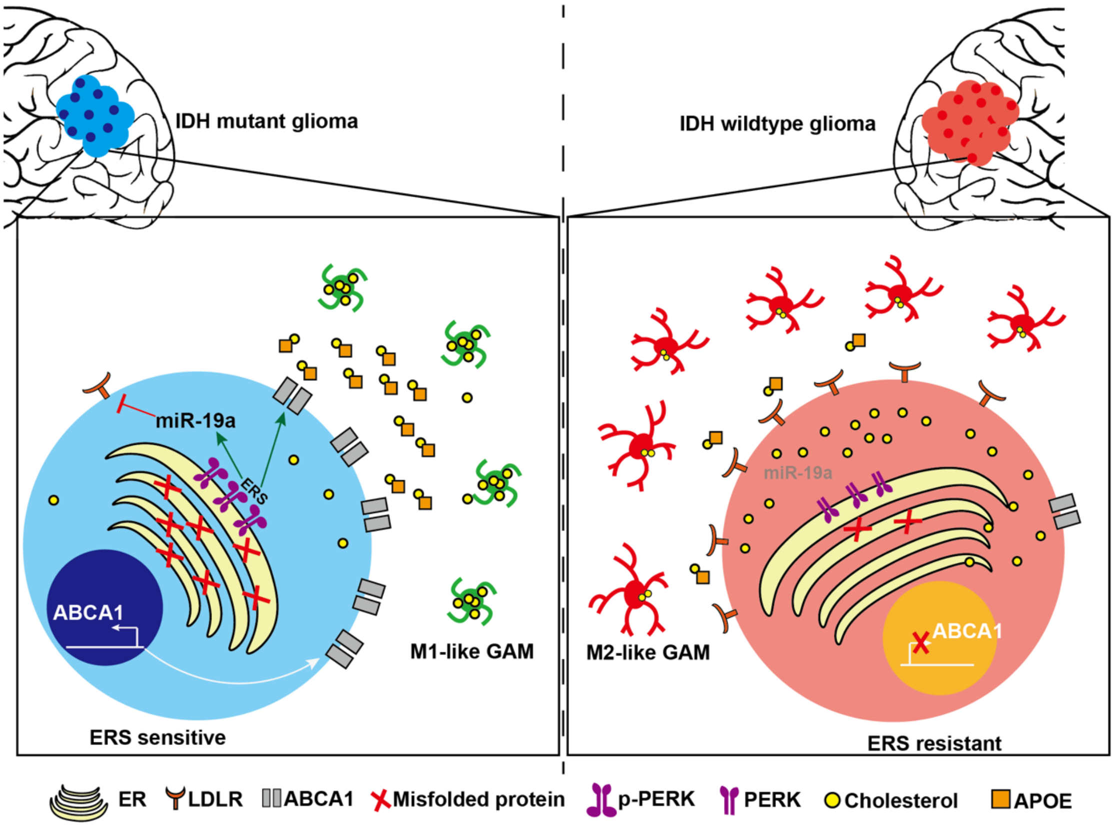

Figure S9. Working model illustrating how gliomal IDH genotypes regulate GAM polarization via the miR-19a/LDLR axis-mediated cholesterol export in a PERK-dependent manner.

Figure S10

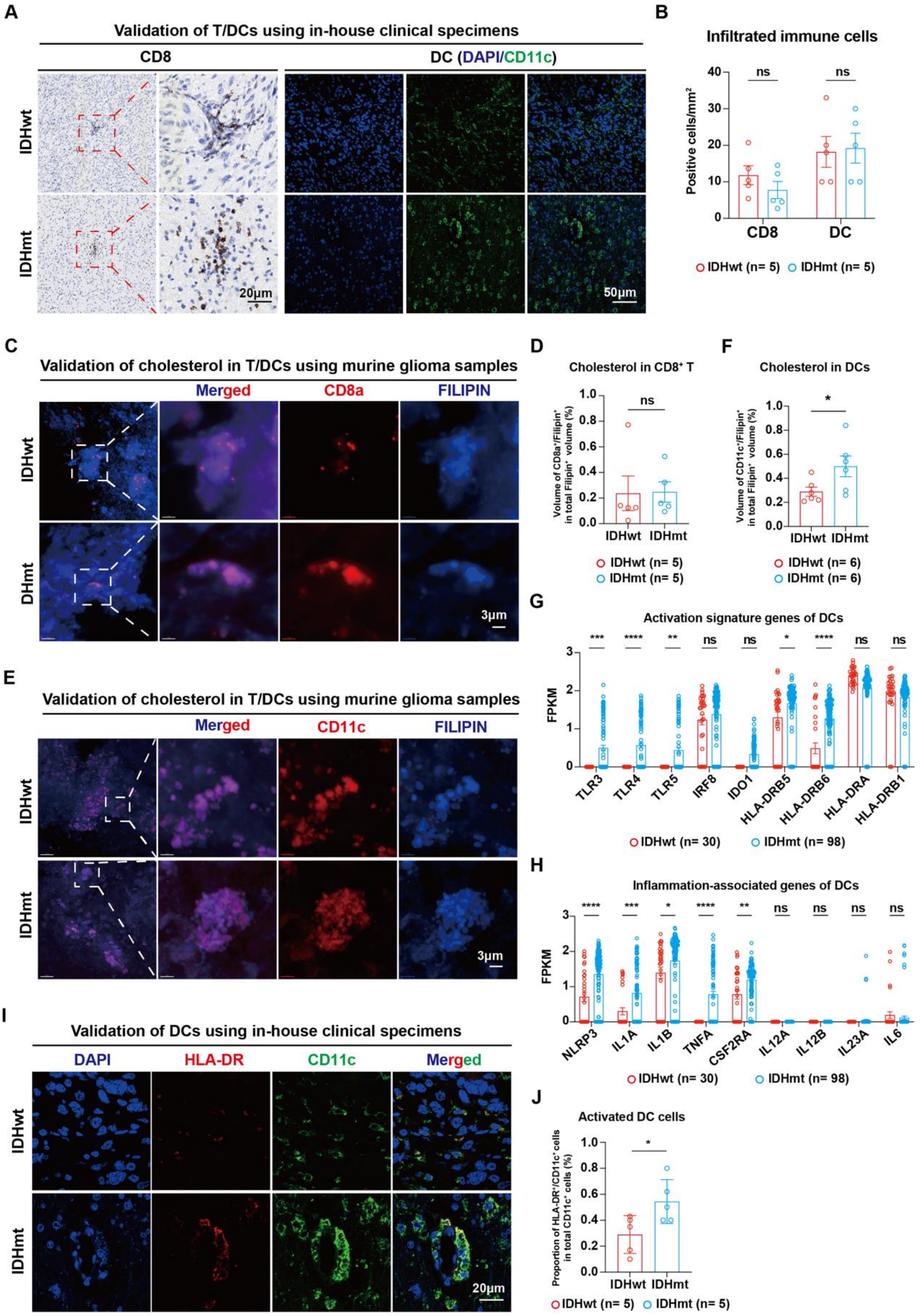

Figure S10. Potential impact of cholesterol accumulation in the infiltrated DCs and CD8^{+} T cells between IDHmt and IDHwt gliomas. (**A-B**) Representative

images (A) and quantification (B) of IHC and IF staining for CD8$^+$ and CD11c$^+$ in human glioma samples, respectively. (**C**) Representative images of IF staining for CD8a (red) and Filipin staining (blue) in a syngeneic glioma mouse model. (**D**) Quantification of the percentage of Filipin$^+$/CD8a$^+$ colocalized volume in total Filipin$^+$ volume. (**E**) Representative images of IF staining for CD11c$^+$ (red) and Filipin staining (blue) in a syngeneic glioma mouse model. (**F**) Quantification of the percentage of Filipin$^+$/CD11c$^+$ colocalized volume in total Filipin$^+$ volume. (**G-H**) Expression of genes associated with DC activation (G) and inflammation (H) using published scRNA-seq datasets. (**I-J**) Representative images (I) and quantification (J) of IF staining for CD11c$^+$ and HLA-DR$^+$ in human glioma samples. Data are shown as the means ± SEM.. Data are shown as the means ± SEM. Statistical significance is determined by the two-tailed Student`s t-test (B, D, F, and J) or two-way ANOVA (G-H), ns, $p>0.05$; *, $p< 0.05$; **, $p< 0.01$; ***, $p< 0.001$; ****, $p< 0.0001$.

Figure S11

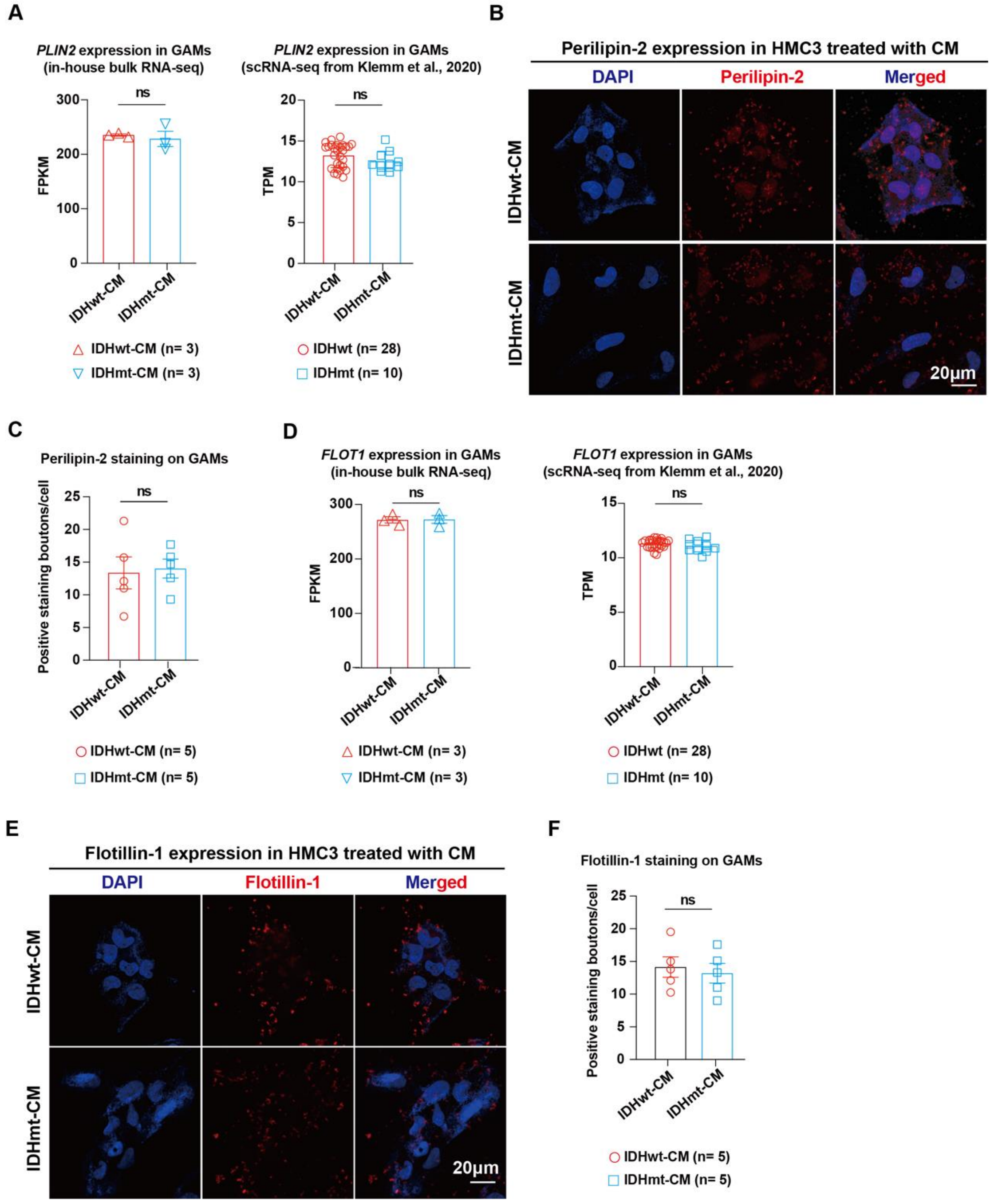

Figure S11. Expression of lipid-associated molecules, PLIN2 and FLOT1, in GAMs. (**A**) Comparison of PLIN2 expression in GAMs using in-house bulk RNA-seq and published scRNA-seq datasets. (**B-C**) Representative images (B) and quantification (C) of IF staining for Perillinpin-2 in HMC3 cells treated with IDHwt-CM or IDHmt-CM. (**D**) Comparison of FLOT1 expression in GAMs using

in-house bulk RNA-seq and published scRNA-seq datasets. (**E-F**) Representative images (E) and quantification (F) of IF staining for Flotillin-1 in HMC3 cells treated with IDHwt-CM or IDHmt-CM. Data are shown as means ± SEM. Statistical significance is determined by the two-tailed Student`s t-test, ns, $p>0.05$.

Figure S12

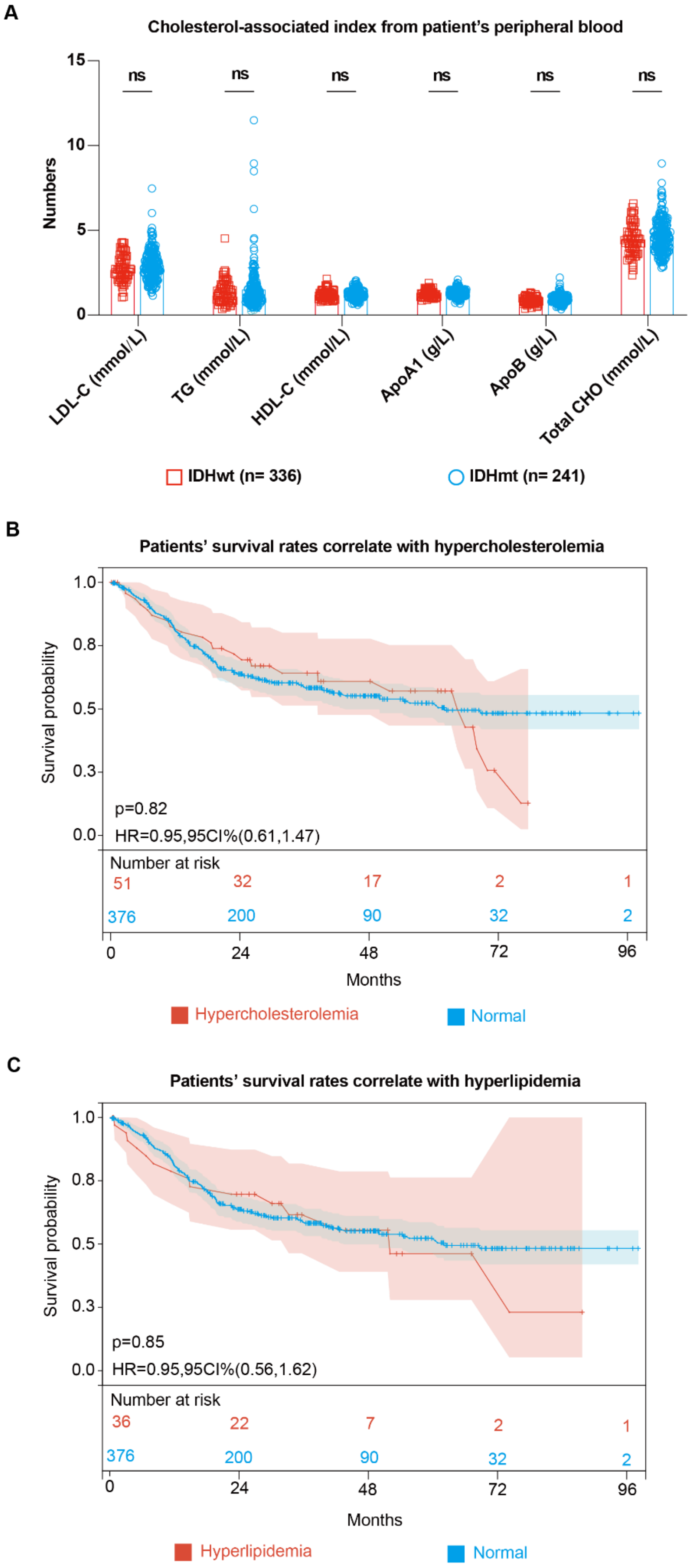
A
Cholesterol-associated index from patient's peripheral blood
Numbers
15
10
5
0
ns
ns
ns
ns
ns
ns
LDL-C (mmol/L)
TG (mmol/L)
HDL-C (mmol/L)
ApoA1 (g/L)
ApoB (g/L)
Total CHO (mmol/L)
IDHwt (n= 336)
IDHmt (n= 241)
B
Patients' survival rates correlate with hypercholesterolemia
Survival probability
1.0
0.8
0.5
0.3
0.0
p=0.82
HR=0.95,95CI%(0.61,1.47)
Number at risk
51
32
17
2
1
376
200
90
32
2
0
24
48
72
96
Months
Hypercholesterolemia
Normal
C
Patients' survival rates correlate with hyperlipidemia
Survival probability
1.0
0.8
0.5
0.3
0.0
p=0.85
HR=0.95,95CI%(0.56,1.62)
Number at risk
36
22
7
2
1
376
200
90
32
2
0
24
48
72
96
Months
Hyperlipidemia
Normal

Figure S12. Insignificant correlation of hypercholesterolemia/hyperlipidemia with the prognosis of glioma patients. (**A**) Comparison of in-house peripheral cholesterol indexes in glioma patients (IDHmt: IDHwt= 241: 336). Data are shown as mean ± SEM. Statistical significance is determined by the one-way ANOVA, ns, $p>0.05$. (**B**) Kaplan-Meier overall survival (OS) curves for glioma patients with or without hypercholesterolemia. (**C**) Kaplan-Meier overall survival (OS) curves for glioma patients with or without hyperlipidemia.
